# Tunable Aryl Alkyl Ionic Liquid Supported Synthesis of Platinum Nanoparticles and Their Catalytic Activity in the Hydrogen Evolution Reaction and in Hydrosilylation

**DOI:** 10.3390/molecules28010405

**Published:** 2023-01-03

**Authors:** Dennis Woitassek, Till Strothmann, Harry Biller, Swantje Lerch, Henning Schmitz, Yefan Song, Stefan Roitsch, Thomas Strassner, Christoph Janiak

**Affiliations:** 1 Institut für Anorganische Chemie und Strukturchemie, Heinrich-Heine-Universität Düsseldorf, 40204 Düsseldorf, Germany; 2 Physikalische Organische Chemie, Technische Universität Dresden, 01062 Dresden, Germany; 3 Institut für Physikalische Chemie, Universität zu Köln, 50939 Köln, Germany

**Keywords:** platinum, nanoparticles, hydrogen evolution reaction, ethylene glycol, microwave heating, ionic liquid, tunable aryl alkyl ionic liquid, hydrosilylation

## Abstract

Tunable aryl alkyl ionic liquids (TAAILs) are ionic liquids (ILs) with a 1-aryl-3-alkylimidazolium cation having differently substituted aryl groups. Herein, nine TAAILs with the bis(trifluoromethylsulfonyl)imide anion are utilized in combination with and without ethylene glycol (EG) as reaction media for the rapid microwave synthesis of platinum nanoparticles (Pt-NPs). TAAILs allow the synthesis of small NPs and are efficient solvents for microwave absorption. Transmission electron microscopy (TEM) shows that small primary NPs with sizes of 2 nm to 5 nm are obtained in TAAILs and EG/TAAIL mixtures. The Pt-NPs feature excellent activity as electrocatalysts in the hydrogen evolution reaction (HER) under acidic conditions, with an overpotential at a current density of 10 mA cm^−2^ as low as 32 mV vs the reversible hydrogen electrode (RHE), which is significantly lower than the standard Pt/C 20% with 42 mV. Pt-NPs obtained in TAAILs also achieved quantitative conversion in the hydrosilylation reaction of phenylacetylene with triethylsilane after just 5 min at 200 °C.

## 1. Introduction

**Figure 1:**
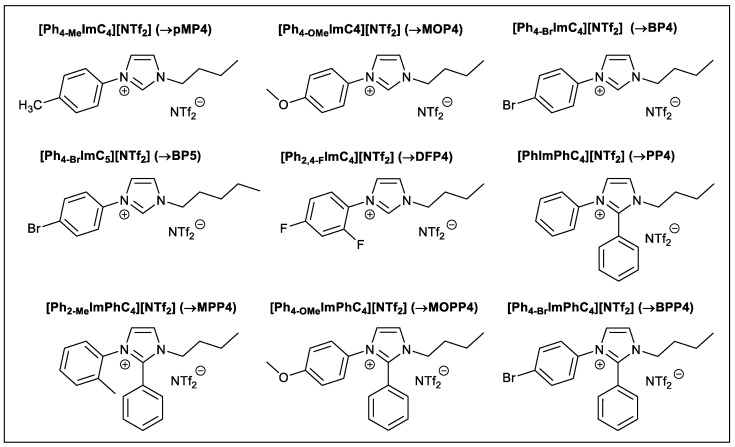
TAAILs utilized in this work with bis(trifluoromethylsulfonyl)imide, [NTf_2_]^−^ as anion and the sample designation (in parentheses) for the (TAAIL)Pt-NPs.

A consistently high interest in heterogeneous catalysis is dedicated to Pt nanostructures and their alloys, which are known for their high catalytic activity in oxidation, hydrogenation, hydrosilylation, and electrocatalysis reactions [1,2,3,4,5,6,7,8,9]. The large surface area in relation to their volume gives nanoparticles (NPs) a high mass-based activity. The catalytic activity and stability of nanoparticles are further determined by their size, composition, shape, surface structure, protection, and surface accessibility [1,10,11]. To prevent coalescence, agglomeration, or Ostwald ripening of NPs stabilizing capping ligands, surfactants or polymers are crucial for utilizing NPs [12,13,14,15,16,17]. One group of convenient nanoparticle stabilizers are ionic liquids (ILs), which can also function as reaction media for NP synthesis [12,18,19,20,21]. In addition, ILs are excellent solvents for microwave reactions due to their high absorptivity of microwave irradiation which allows for a combination of fast and homogenous microwave heating with the stabilizing effects of ILs, resulting in an effective way to produce small NPs [21,22,23,24]. Tunable aryl alkyl ionic liquids (TAAILs) are a newer class of ILs, which contain an *N*-aryl group as well as an *N*-alkyl chain on the imidazole ring (Figure 1). Both substituents can be tailored to influence the reaction environment and the resulting properties of NPs [18,21,24]. Another widespread method for the synthesis of metal NPs is the polyol process using ethylene glycol (EG) as a solvent and as a stabilizer [25,26,27]. EG is also suitable for microwave heating as it offers a high boiling point and a strong absorptivity of microwave irradiation [28]. Generally, size and shape control in EG-mediated synthesis is achieved by adding surfactants [25,29]. The addition of sodium hydroxide to EG allows a surfactant-free approach, which also enables reliable size control [30,31,32]. Although polyols are well-researched solvents and reducing agents for NP synthesis, mixtures of ILs and polyols as solvents are less common [33,34,35,36,37,38]. Such mixtures have been used as a solvent system for the synthesis of various mono- and multimetallic NPs and the sonochemical synthesis of different M-NPs [33,34,35,36,37,38], but not yet for Pt-NPs in a microwave setting. Moreover, polyol and IL mixtures are utilized for biomolecule extraction and as electrolytes for electrodeposited metal nanostructures [39,40,41,42].

In this article, we present the microwave-assisted synthesis of Pt-NPs in TAAILs with the 1-aryl-3-alkyl- imidazolium cation and novel TAAILs that contain an additional phenyl group on the imidazolium C2-position [43]. As reaction media, the TAAILs are used individually and in combination with EG.

The obtained Pt-NPs were tested for their activity toward the electrochemical hydrogen evolution reaction (HER) and toward the hydrosilylation of phenylacetylene with triethylsilane. It is known that Pt compounds can form catalytically active Pt-NP species in situ in ionic liquids [44,45,46]. Therefore, we also examined the use of potassium hexachloridoplatinate(IV) (K_2_PtCl_6_) dispersed in an EG/TAAIL phase for hydrosilylation without a preceding NP separation.

## 2. Results and Discussion

### 2.1. Tunable Aryl Alkyl Ionic Liquids (TAAILs)

All 1-aryl-3-alkylimidazolium bis(trifluoromethylsulfonyl)imide ([Ph_x_ImC_4_][NTf_2_]) and 1-aryl-2-aryl-3-alkylimidazolium bis(trifluoromethylsulfonyl)imide ([Ph_x_ImPhC_4_][NTf_2_]) TAAILs which were used for the Pt-NP synthesis are presented in Figure 1. With the exception of [Ph_4-Br_ImC_5_][NTf_2_], which contains an *n*-pentyl substituent, only *n*-butyl (C_4_) substituents were present as alkyl groups. The [NTf_2_]^−^ anion has been chosen because it induces a low melting temperature, high inertness, and hydrophobicity to the IL, with the latter being important to prevent water uptake that could cause the deactivation of the catalyst during hydrosilylation. It has also been shown that ILs containing the [NTf_2_]^−^ anion are most suitable for hydrosilylation reactions [45,47]. The synthesis and characterization of [Ph_4-Br_ImPhC_4_][NTf_2_] and its precursors can be found in the Supplementary Materials while the synthesis of the other TAAILs has been described by Strassner et al. before [43,48,49]. NMR spectra of all TAAILs can be found in Sections S2.1 and S2.2 (Supplementary Materials).

TAAIL anion purity and temperature stability have been examined by ion chromatography (IC) and thermogravimetric analysis (TGA), respectively, with the results shown in Table 1 and in Section S2.3 and Figure S13, Section S2.4. Anion purities over 92% and IL purities of at least 97% were achieved, with traces of halogenides as residual anions remaining from the ion exchange. All ILs are stable up to at least 390 °C under a nitrogen atmosphere, similar to other ILs and TAAILs containing [NTf_2_]^−^ anions [24,48,49,50,51], and are, thus, suitable solvents for microwave reactions at 200 °C.

### 2.2. Synthesis and Characterization of Pt-NPs in TAAILs

**Scheme 1:**
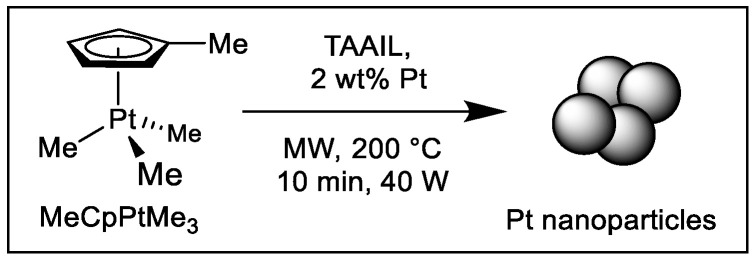
Reaction conditions for the microwave-assisted synthesis of (TAAIL)Pt-NPs. The amount of precursor was set to achieve 1 or 2 wt% Pt-NP in IL.

Scheme 1 presents the general synthetic approach of (TAAIL)Pt-NPs via microwave heating and follows the synthesis procedure of Pt-NPs in TAAILs previously reported [21,22,24]. Microwave conditions offer fast, uniform heating, resulting in homogenous Pt-NPs and ILs providing a stable, fast heating media and acting as stabilizers for the formed NPs. The Pt precursor (η^5^-methylcyclopentadienyl)trimethylplatinum(IV) (MeCpPtMe_3_) can be decomposed at mild reaction conditions without additional reducing agents to Pt-NPs [16,22]. Together with the Pt-NPs, only volatile side products are obtained which are removed from the decomposition of MeCpPtMe_3_, resulting in a contaminant-free Pt-NP dispersion [52]. The Pt-content was set to one or two weight percent (wt%) in the TAAIL dispersion.

**Figure 2:**
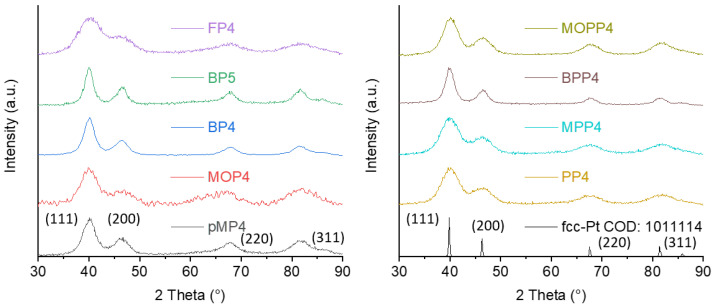
PXRD patterns of synthesized (TAAIL)Pt-NPs (cf. Figure 1 and Table 2). All obtained reflexes match the simulation for face-centered cubic, fcc-Pt with its indexed reflections (Crystallographic open database, COD fcc-Pt: 1011114).

The microwave reaction was carried out at 200 °C for 10 min. Afterward, the obtained black Pt-NP dispersion was washed several times with acetonitrile (ACN), separated by centrifugation, and dried in a vacuum, giving a nearly quantitative yield of Pt-NPs. Microwave-assisted heating of metal precursors in IL dispersions results in small M-NP sizes as was shown for Ir-NPs, Ru-NPs [24], and Pt-NPs [21,22]. Fast microwave heating and efficient energy absorption by the IL lead to rapid decomposition of the metal precursor and a high nucleation rate of metal NPs. These metal NPs themselves absorb microwave radiation very efficiently, leading to “hot spots” with a further locally increasing temperature [53,54]. In Figure 2, powder X-ray diffraction (PXRD) patterns of the Pt-NP samples show reflexes matching crystalline fcc-Pt. The Pt-NP sizes have been determined as crystallite sizes from the peak widths in the PXRD patterns with the Scherrer equation and as particle sizes from transmission electron microscopy (TEM) images. These values are listed in Table 2. The crystallite sizes from the Scherrer equation (see Section 3 Materials and Methods) range from 3 nm to 5 nm.

The particle sizes from TEM (Table 2) are similar to the calculated crystallite sizes from PXRD, indicating the formation of isolated nanocrystals. Compared to Pt-NPs synthesized in TAAILs previously [21], the crystallite sizes obtained here for (TAAIL)Pt-NPs are very similar and seem independent of the TAAIL or of the targeted wt% Pt in IL (Table S3 and Figure S14 in Section S3). Pt-NPs which were synthesized from MeCpPtMe_3_ in the TAAILs [Ph_2-Me_ImC_4/5/9_][NTf_2_], [Ph_2,4-Me_ImC_9_][NTf_2_] and [Ph_4-OMe_ImC_5_][NTf_2_] had a particle size of 2 to 5 nm [21]. When the Pt-NPs were deposited on reduced graphene oxide from the TAAILs, the particle size was found between 2 to 6 nm [21]. It is sometimes argued that metal NPs and imidazolium ILs form metal *N*-heterocyclic carbene (NHC) complexes on the NP surface [55,56]. For the NHC-metal complex formation, the C2 position between the imidazolium nitrogen atoms is deprotonated. In the 1-aryl-2-aryl-3-alkylimidazolium TAAILs [Ph_x_ImPhC_4_][NTf_2_], the C2 position of the imidazolium core is blocked and the observed (TAAIL)Pt-NP particle size is not affected. This speaks against a carbene formation in the utilized TAAILs (although carbene formation in the C4 or C5 positions cannot be fully ruled out).

**Figure 3:**
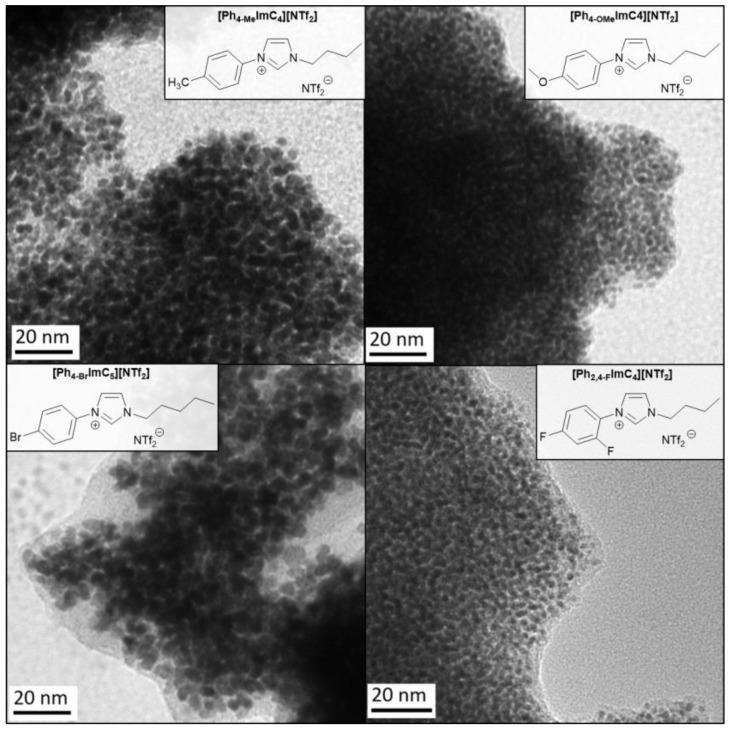
TEM images of (TAAIL)Pt-NPs obtained in the (non-C2-substituted) TAAILs [Ph_4-Me_ImC_4_][NTf_2_] (MP4), [Ph_4-OMe_ImC_4_][NTf_2_] (MOP4), [Ph_4-Br_ImC_5_][NTf_2_] (BP5) and [Ph_2,4-F_ImC_4_][NTf_2_] (DFP4).

**Figure 4:**
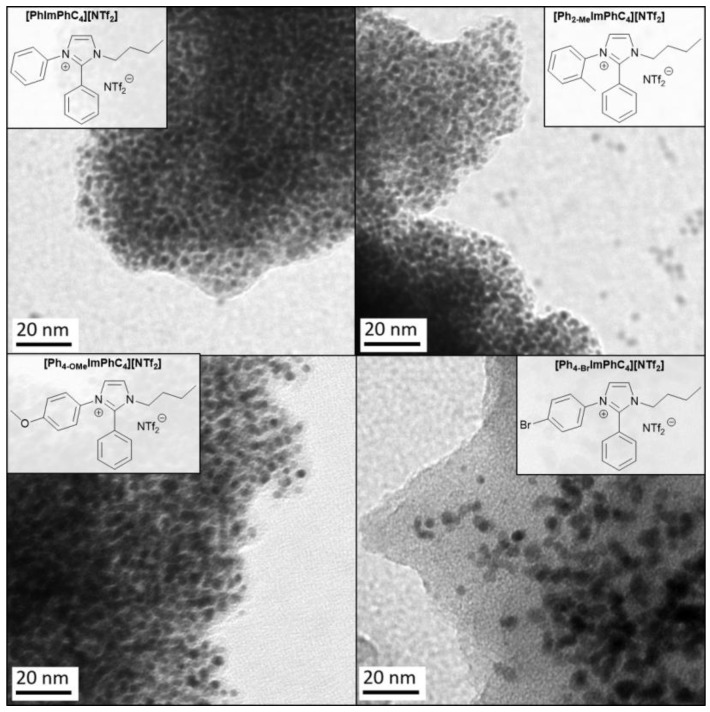
TEM images of (TAAIL)Pt-NPs obtained in the TAAILs [PhImPhC_4_][NTf_2_] (PP4), [Ph_2-Me_ImPhC_5_][NTf_2_] (MPP4), [Ph_4-OMe_ImPhC_4_][NTf_2_] (MOPP4) and [Ph_4-Br_ImPhC_4_][NTf_2_] (BPP4).

TEM images of Pt-NPs obtained in TAAILs are collected in Figure 3 and Figure 4 for non-C2- and C2-substituted TAAILs, respectively. All [Ph_x_ImC_4_][NTf_2_] and [Ph_x_ImPhC_4_][NTf_2_] samples exhibit dense Pt-NP aggregates with the edges showing a thin layer from residual TAAILs that may hold the Pt-NPs together. This dense aggregation after microwave-assisted synthesis in ILs was not only seen for Pt-NPs before [21,22], but also for Ir-NPs and Ru-NPs [24]. The methoxy, bromo, or fluoro functionalization of the *N*-aryl group in the TAAILs of the (TAAIL)Pt-NPs MOP4, MOPP4, BP5, BPP4, and DFP4 does not affect the particle size or aggregation observed with TEM when compared to the alkyl-substituted aryl groups. This finding is similar to those observed for Pt-NPs in TAAILs before [21]. Only in the bromo-functionalized TAAILs of BP5 and BPP4 smaller aggregates with larger, more isolated NPs are seen, together with a larger amount of residual TAAIL. Noteworthy, all samples have been thoroughly washed with ACN until a clear centrifugate could be achieved. We conclude that the NP-adherent IL layer is difficult to remove and the bromo derivatives BP5 and BPP4 may feature an even lower solubility in ACN. TEM images of the other samples can be found in Section S3, Figures S16–S24.

### 2.3. Synthesis and Characterization of Pt-NPs in EG/TAAIL Mixtures

**Scheme 2:**
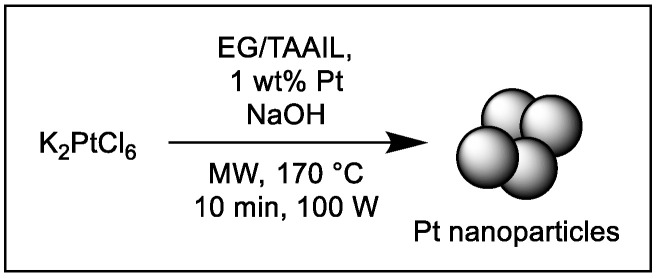
Reaction conditions for synthesizing (EG/TAAIL)Pt-NPs via microwave heating in EG/TAAIL mixtures. The amount of precursor used has been chosen to achieve 1 wt% Pt-NP in EG/IL.

The synthesis of (EG/TAAIL)Pt-NPs is depicted in Scheme 2. This method is a modified version of the surfactant-free polyol process presented by Quinson et al. [30] with potassium hexachloridoplatinate(IV), K_2_PtCl_6_ as Pt precursor, and the addition of 10, 25, 50, or 75 wt% IL to EG, that is, using a 9/1, 3/1, 1/1 or 1/3 EG/TAAIL mass ratio, respectively. The Pt-content was set to 1 wt% Pt in EG/IL. The reaction was carried out in a glass vial under microwave irradiation with a reaction temperature of 170 °C. Reactions with MeCpPtMe_3_ as a metal source were unsuccessful; even at 195 °C, no Pt-NP formation was observed. K_2_PtCl_6_ was chosen as Pt precursor instead because it is a common Pt source for the Pt-NP synthesis [57,58] and can be effectively reduced by EG at the temperature of 170 °C.

The addition of NaOH is an established procedure to limit the growth of NPs in the polyol process. The effect depends on the ratio between precursor and NaOH and is suggested to derive from the coordination of hydroxide ions onto the NP surface [30].

The synthesis of (EG/TAAIL)Pt-NPs without NaOH produced significantly larger particles as was already observed before for M-NP formation (M = Pt, Ir) in EG [30,59]. Quinson et al. have shown that a NaOH/H_2_PtCl_6_ molar ratio of ~12/1 produces Pt-NPs with a size of ~2 nm in neat EG [30], which is why this ratio was also used in this work.

**Figure 5:**
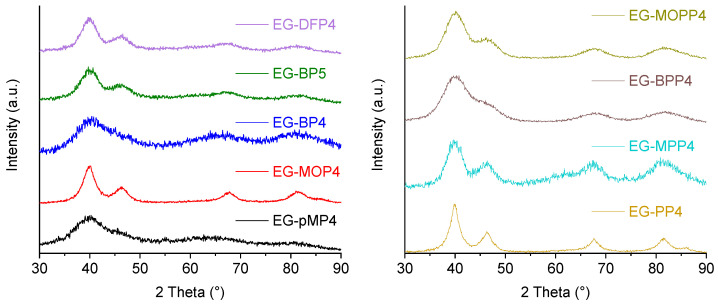
PXRD patterns of (EG/TAAIL)Pt-NPs(cf. Figure 1 and Table 3). All obtained reflexes match the simulation for fcc-Pt and its indexed reflections (cf. Figure 2).

After microwave heating, the resulting black dispersions were washed multiple times with ACN until a clear solution after centrifugation could be separated. The remaining sodium salts were removed afterward by washing twice with methanol. The (EG/TAAIL)Pt-NPs were dried in a vacuum and obtained quantitative yields, as the (TAAIL)Pt-NPs above. PXRD patterns in Figure 5 confirm the nanocrystallinity of the platinum particles. The crystallite sizes, given in Table 3, were determined from the peak widths in the PXRD patterns via the Scherrer equation. The PXRD patterns exhibit no reflexes that could be attributed to sodium or potassium chloride residues. Higher amounts of IL in EG (25, 50, and 75 wt%) led to larger crystallite sizes (Table S4 and Figure S15). At very low EG/IL-ratios (1/9, 90 wt% IL), no Pt-NP formation was observed anymore from K_2_PtCl_6_, presumably due to the low concentration of the EG-reducing agent.

**Figure 6:**
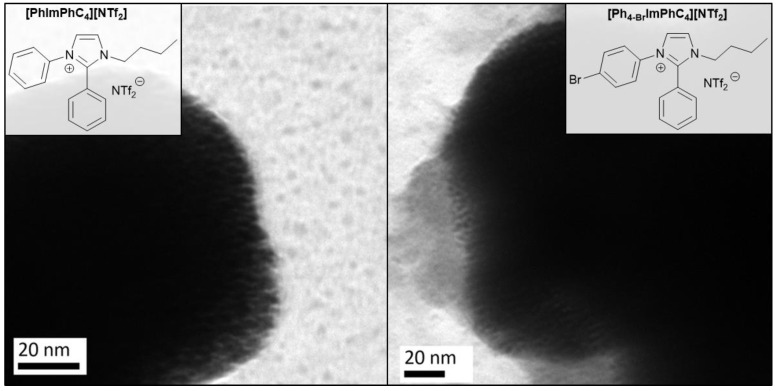
TEM images of (EG/TAAIL)Pt-NPs obtained in [PhImPhC_4_][NTf_2_] (PP4) and [Ph_4-Br_ImPhC_4_][NTf_2_] (BPP4), respectively. TEM images of the other samples can be found in Figures S25–S33.

The crystallite sizes calculated from PXRD patterns, and the particle sizes observed from the TEM images are given in Table 3. Comparable to the particles in pure TAAIL, the (EG/TAAIL)Pt-NPs all show similar crystallite sizes between 2 nm and 5 nm. TEM images of two (EG/TAAIL)Pt-NP samples are given in Figure 6. In general, the particles form large and dense agglomerates of several 100 nm in size. Different from the Pt-NPs in neat TAAILs which were depicted in Figure 4, the individual Pt-NPs in EG/TAAIL can hardly be differentiated anymore. This indicates a lower particle-separating effect of EG in comparison to ILs. With an excess of EG over TAAIL, the outer solvent layer adherent to the aggregated NPs is smaller and less regular compared to neat TAAIL.

### 2.4. Hydrogen Evolution Reaction (HER)

HER is one of the half-reactions for water splitting to generate molecular hydrogen for the storage of renewable wind or solar electricity [60,61]. Platinum is known as a highly active electrocatalyst for this reaction in acid media, yet its scarcity and high cost hinder its deployment in large-scale industrial applications [62,63]. The electrocatalytic activity towards HER of (TAAIL)Pt-NPs and (EG/TAAIL)Pt-NPs in 0.5 mol L^−1^ sulfuric acid was investigated. Activation of the samples was achieved by cyclovoltammetry (see Section 3.3 Materials and Methods). The samples that showed an overpotential of less than 60 mV at 10 mA cm^−2^ after activation were also subjected to a stability test. As reference material, commercially available Pt on carbon (Pt/C 20 wt%) was used and its electrochemical data agreed with literature reports [64,65].

**Figure 7:**
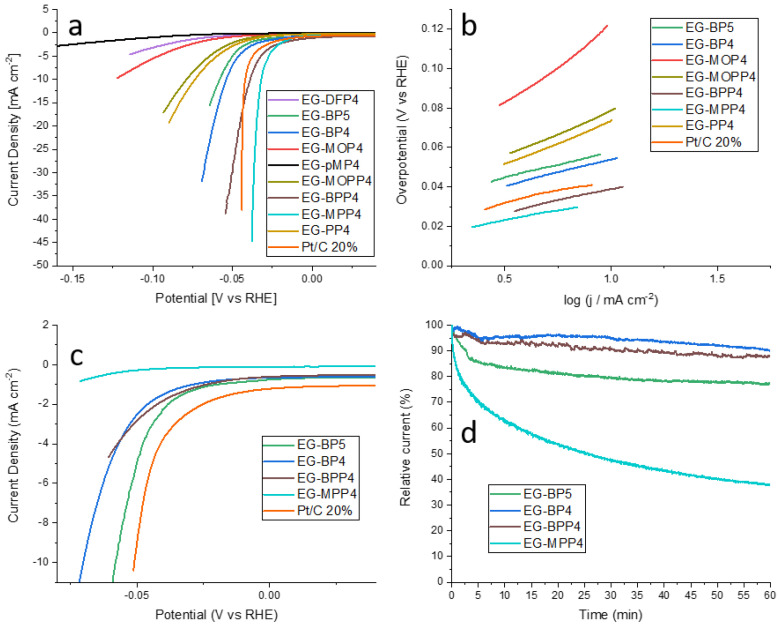
HER polarization curves of (EG/TAAIL)Pt-NPs (**a**), Tafel plots of the samples that reached an overpotential of 10 mA cm^−2^ (**b**), polarization curves after a 1000 CV stability test (**c**), and the chronoamperometric current loss of selected samples (**d**).

Figure 7a displays the polarization curves of the (EG/TAAIL)Pt-NP samples and the reference material after activation. The electrochemical parameters are summarized in Table 4. EG-MPP4 reached the lowest overpotential of 32 mV, outperforming Pt/C 20 wt% with an overpotential of 42 mV. The overpotential of EG-MPP4 was similar to those of single-atom Pt-Catalysts (Pt_1_/OLC and ALD50Pt/NGNs) and Pt-Ni nanowires (Pt_3_Ni_2_ NWs-S/C) with overpotentials of 38, 50 and 27 mV, respectively [66,67,68]. Additionally, EG-BPP4, with an overpotential of 39 mV, still performed slightly better than the reference material Pt/C 20 wt%. EG-BP4 and EG-BP5 both have somewhat higher overpotentials of 54 mV and 58 mV, respectively. The remaining samples showed fairly high overpotentials or did not reach the necessary current. (EG/TAAIL)Pt-NP probes with TAAILs substituted at the C2 position display lower overpotentials and also bromo functionalization the *N*-aryl group produces NPs with lower overpotentials. (TAAIL)Pt-NPs have also been analyzed electrochemically but are mostly inactive, with most samples not reaching a current density of 10 mA cm^−2^ under the measurement conditions (polarization curves are displayed in Section S4). In general, (EG/TAAIL)Pt-NP samples performed better than those in TAAIL alone.

Figure 7b displays the Tafel plots based on the kinetically controlled areas at low overpotentials for those samples that reached a current density of 10 mA cm^−2^. The Tafel slope describes the increase of the overpotential required for a ten-fold increase of the current density [69]. A low Tafel slope is a good indicator of an effective electrocatalyst [65]. The (EG/TAAIL)Pt-NP sample with the lowest overpotential, EG-MPP4, also has the lowest Tafel slope of 20 mV dec^−1^. All samples with brominated TAAILs, that is EG-BPP4, EG-BP4, and EG-BP5, give similar Tafel slopes of 24 mV dec^−1^, 27 mV dec^−1^ and 26 mV dec^−1^, respectively, similar to Pt/C 20% with 25 mV dec^−1^. Similar Tafel slopes have also been reported for single-atom Pt catalysts (Pt_1_/OLC and ALD50Pt/NGNs) [66,68]. Much higher Tafel slopes are seen for EG-PP4 with 44 mV dec^−1^ and EG-MOPP4 with 46 mV dec^−1^, with EG-MOP4 having the highest value of 78 mV dec^−1^. The long-term stability of the catalysts was verified via a cyclic voltammetry (CV) durability test comprising 1000 CV cycles. The polarization curves after the stability tests are plotted in Figure 7c. EG-BP4 revealed a significant decrease in activity and reached an overpotential of 70 mV. All other samples ended with larger activity losses and did not reach 10 mA cm^−2^ anymore under the measurement conditions, including EG-MPP4, which was the most active (EG/TAAIL)Pt-NP prior to the stability test. Chronoamperometry has been performed as an alternative stability test for the samples that also underwent CV stability tests. The relative current density losses over time are displayed in Figure 7d. Similar to CV stability tests, EG-BP5, EG-BP4, and EG-BPP4 all show a moderate activity loss. The activity of EG-BP5, the sample with the lowest activity loss during CV stability tests, lost almost 15% of its activity within 3 min but stays more stable afterward with a total current loss of 22% after 60 min. In contrast to CV stability tests, both EG-BP4 and EG-BPP4 show slightly less activity loss than EG-BP5, with 5% reduced activity after 7 min and 10% and 12% after 60 min, respectively. All three samples exhibit similar long-term behavior, after the initial activity changes within the first minutes. EG-MPP4 degenerates much more rapidly, losing over 60% activity within 60 min, and is in agreement with the CV stability test.

### 2.5. Hydrosilylation Reaction

**Scheme 3:**
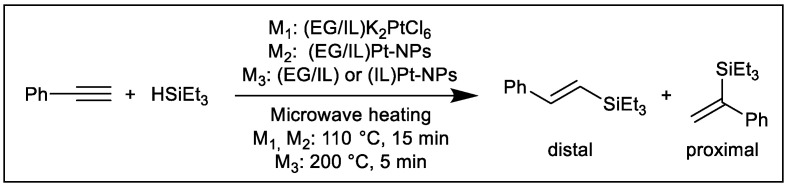
Hydrosilylation of phenylacetylene with triethylsilane in the presence of Pt-catalysts with three methods (labeled M1 to M3). The proximal triethyl(1-phenylvinyl)silane and distal triethyl(styryl)silane [triethyl(2-phenylvinyl)silane] products are obtained.

The hydrosilylation of phenylacetylene with triethylsilane, has been chosen as a proof of principle to determine the catalytic activity of Pt-NPs in conjunction with ionic liquids. Hydrosilylation is of high importance for modern silicone chemistry for the addition of Si-H bonds to C-C multiple bonds [70,71,72]. For industrial applications of the hydrosilylation reaction, almost exclusively noble-metal catalysts containing Ir, Ru, Pd, or the Speier and Karstedt Pt catalysts, are employed [73,74]. Until now, the Chalk-Harrod mechanism is the most accepted mechanism for heterogeneous hydrosilylation [70,71,72,75], which we assume to apply to our IL/NP system as well. The terminal acetylene can be hydrosilylated at both carbon atom positions, resulting in a proximal and distal product. On the lab scale, microwave conditions offer a fast, energy-efficient alternative compared to conventional oil bath heating. We chose three different microwave-assisted methods to investigate the catalytic activity of the catalyst systems as sketched in Scheme 3. Method 1 describes a reaction at 110 °C for 15 min consisting of an EG/TAAIL liquid, K_2_PtCl_6_, and the substrate phase. Pt-salts in IL have already been shown as promising catalyst systems for the hydrosilylation reaction [44,45,46]. For method 2 and method 3, the (EG/TAAIL)Pt-NP and (TAAIL)Pt-NP samples have been heated with the substrate phase at 110 °C for 15 min and 200 °C for 5 min, respectively. The conversions of the substrates and the ratios between the distal and proximal products were determined by ^1^H NMR spectroscopy and gas chromatography coupled with mass spectrometry (GC-MS) (see Sections S5.2 and S5.4, respectively).

In Table 5 and Table 6, the catalytic conversions with selected catalyst samples and reference catalysts are summarized, respectively (see Table S5 for the full list). The IL-containing probes together with microwave heating led to a significant reduction in reaction time to achieve high conversions compared to literature reports with conventional thermal heating [44,45,75,76,77,78,79,80]. The catalyst derived from EG/IL K_2_PtCl_2_ with method 1 generally achieved quantitative conversion after 15 min, with some exceptions for non-C2-substituted TAAILs (Section S5.1). Distal/proximal product ratios were between two to three. EG/TAAIL mixtures without Pt catalyst and (TAAIL)K_2_PtCl_6_ showed no product formation (see Section S5). The sample (EG)K_2_PtCl_6_ without TAAIL gave a conversion of only 50%. It is assumed in the literature that the catalytically active Pt species from Pt-salts in IL are in situ formed Pt-NPs [44,45,46]. Yet, the (EG/TAAIL)Pt-NP samples used with method 2 (110 °C for 15 min) yielded significantly less conversion (at most 38 to 75%) than the reaction with K_2_PtCl_6_ after method 1. The distal/proximal ratios range from 3.1 to 3.6. (TAAIL)Pt-NPs according to method 2 as well as reference Pt-NPs obtained in the IL [BMIm][NTf_2_] [21] did not show any conversion at all.

We have shown before that an increased temperature can lead to quantitative conversions after just 5 min [81]. We prepared a similar approach for our samples with method 3. In general, the conversions were significantly higher while often reaching quantitative yields for (EG/TAAIL)Pt-NPs and (TAAIL)Pt-NPs. A Pt-free reaction resulted in no conversion while K_2_PtCl_6_ and Pt-NPs synthesized in [BMIm][NTf_2_] also gave high yields (Table S5). In general, the distal/proximal ratios detected are lower than for the other two methods, with a minimum of 1.5 and an average of two. GC-MS generally resulted in slightly reduced distal/proximal ratios compared to the ratios determined by ^1^H NMR (see Table S5 for the full list).

In comparison to literature results for Pt-NPs collected in Table 6 [44,45,75,76,77,78,79,80], the catalysis following method 1 and 3 resulted in quantitative yields in remarkably shorter reaction times. However, both methods only achieved the preferred formation of the distal product with a distal/proximal ratio of ~2–3 while reference reactions can achieve stronger preferences for one specific product with distal/proximal ratios as low as 0.3 [75] or as high as 9.0 [78]. Many Pt-NP catalysts in the literature yielded d/p ratios between 3.3 and 9.0. Only Pt_1_/NaY and C-Pt/ImIP-2BrB yielded distal/proximal ratios below one (Table 6).

Samples used in method 1 achieved similar conversions in notably shorter reaction time using the same substrate/Pt ratio and temperature as systems with Pt catalysts dispersed in IL reacting 1,1,1,3,5,5,5-heptamethyltrisiloxane with 1-octene [44,45]. The short reaction time and highly diluted dispersion of our (EG/TAAIL) and (TAAIL)Pt catalysts resulted in high turnover frequencies (TOF, highest values under footnote 3 in Table 5), especially for method 1, showing a maximum value of 43,300 h^−1^ for (EG/[Ph_2-Me_ImPhC_4_][NTf_2_])K_2_PtCl_6_. Samples containing the other TAAILs still achieved high TOF values of at least 25,500 h^−1^. Samples used for method 2 and method 3 yielded maximum TOF values of up to 2900 h^−1^ and 12,900 h^−1^, respectively.

Heavy-metal impurities are a challenge for the application of silicones in pharmaceutical or medical products [82,83] and received, for example, high attention for the still contested “breast implant illness” [84]. Contrary to our expectations, graphite furnace atomic absorption spectrometry (GF-AAS) of the majority of the (EG/TAAIL)Pt samples revealed a high Pt leaching into the product solution, up to over 20% of the amount of Pt used for the catalysis (see Table S6). Only (TAAIL)Pt-NP samples from method 3 gave leaching below 1%, which is usually interpreted as no leaching [75,78].

To determine the catalytic stability, the EG/IL phase was recovered for method 1 while for methods 2 and 3, the catalyst was regained after separation from the product via centrifugation, and the recovered catalysts were reused for two additional hydrosilylation reactions. Unexpectedly, all reactions resulted in less conversion compared to the first reaction. The post-mortem TEM images after the third catalysis run of (EG/[Ph_2-Me_ImPhC_4_][NTf_2_])-K_2_PtCl_6_ and (EG/[Ph_4-Br_ImPhC_4_][NTf_2_])-K_2_PtCl_6_ for method 1 and EG-BPP4 for method 2 (Figures S39–S41) show the presence of Pt-NPs. These particles demonstrate a similar degree of aggregation as the (TAAIL)Pt-NPs from which we conclude that, also from EG/TAAIL-K_2_PtCl_6_, platinum nanoparticles form under the catalysis conditions. Zielinski et al. reported hydrosilylation reactions with Pt catalysts dispersed in different ILs and observed a drastic loss of catalytic stability when C=C double bonds were present in the IL [45]. Competitive side reactions between silanes and double bonds were suspected. Catalyst leaching reduces the remaining catalytic activity as well.

In summary, all three presented methods allow the successful hydrosilylation of phenylacetylene with triethylsilane. Method 1 and method 3 can achieve quantitative conversion and high TOF values after the respective reaction time. Reactions carried out after method 2 are not quantitative. Method 1 can be carried out at a temperature of 110 °C, which is more suitable for industrial applications and commonly used in literature experiments [44,45,75]. In the literature, hydrosilylation catalysis is typically performed at temperatures below 110 °C, applying reaction times from 1.3 to 24 h. The reaction times in the literature are longer, but in many cases, only ppm amounts of Pt precursors were used. The reaction times appear to be set to reach the high conversion. The lower reaction temperature makes method 1 superior to method 3. We conclude that the in situ preparation of Pt-NP species in method 1 is, thus, more advantageous in comparison to the independent preparation of Pt-NPs before the catalysis run.

## 3. Materials and Methods

### 3.1. Chemicals and Equipment

All starting materials and solvents were obtained from commercial sources and used as delivered unless mentioned otherwise (Table S1).

Tunable aryl alkyl ionic liquids (TAAIL) were provided by the group of Prof. Dr. Thomas Strassner, Technische Universität Dresden. The synthetic procedure for the TAAIL [Ph_4-Br_ImPhC_4_][NTf_2_] is described in Section S2.1, while the syntheses of the other TAAILs have been documented in the works of T. Strassner [43,48,49]. (η^5^-Methylcyclopentadienyl)trimethylplatinum(IV), MeCpPtMe_3_ was synthesized and characterized using a method described by Xue et al. [22,85].

Transmission electron microscopy (TEM) measurements were carried out with a Zeiss LEO912 (Zeiss, Oberkochen, Germany) at 120 kV accelerating voltage. The microscope features a theoretical spatial resolution of 0.1 nm. The samples were prepared using 200 μm carbon-coated copper grids. 0.05 mL of the NP/IL dispersion was diluted in 0.5 mL acetonitrile (ACN) and one drop of the diluted dispersion was placed on the grid. After 30 min, the grid was washed with 3 mL of ACN and dried in ambient air. The images were analyzed by the program Gatan Microscopy Suite (Version: 3.3, Gatan Inc., Pleasanton, CA, USA) and the particle size distribution was determined from at least 200 individual particles at different positions on the TEM grid within the same magnification.

Powder X-ray diffractograms (PXRDs) were measured at ambient temperature on a Bruker D2-Phaser (Bruker, Billerica, MA, USA) using a flat sample holder and Cu-Kα radiation (λ = 1.54182 Å, 35 kV). The program Diffrac.Eva V4.2 was used to evaluate the PXRD data. Particle sizes were calculated with the Scherrer equation *L* = *K* × λ/(Δ(2*θ*) × cos*θ*) where *L* is the average crystallite size (in nm), *K* the dimensionless shape factor (here 1), λ the wavelength (in nm), Δ(2*θ*) the full width at half maximum (FWHM) in radians and *θ* the Bragg angle (in °).

A CEM-Discover SP microwave reactor (CEM GmbH, Kamp-Lintfort, Germany), with a power range of 0–300 W (±30 W) was used for all microwave reactions.

Thermogravimetric analysis (TGA) was carried out with a Netzsch TG 209 F3 Tarsus (Netzsch, Selb, Germany) in Al crucibles applying a heating rate of 5 K min^−1^ under a nitrogen atmosphere. Determined decomposition temperatures can deviate up to 2 K.

NMR spectra were recorded on a Bruker Avance III-300 (Bruker, Karlsruhe, Germany) and a Bruker Avance III-600 (Bruker, Karlsruhe, Germany) spectrometer (NMR spectra in Sections S2.1 and S5.2). CDCl_3_ was used as a solvent. Chemical shifts were referenced on the residual solvent peak versus TMS (^1^H NMR δ = 7.26 ppm for CHCl_3_, ^13^C NMR δ = 77.16 ppm for CHCl_3_).

Ion chromatography (IC) measurements were performed with a Dionex ICS 1100 instrument (Dionex, Idstein, Germany) with suppressed conductivity detection (chromatograms in Section S2.3). The suppressor (AERS 500, Dionex) was regenerated with an external water module. The system was equipped with the analytical column IonPac AS 22 from Dionex (4 mm × 250 mm) and the corresponding guard column AG 22 (4 mm × 50 mm). The instrument was controlled by Chromeleon^®^ software (Version: 7.1.0.898, Thermo Fisher Scientific GmbH, Dreieich, Germany). The injection volume was 25 μL. The standard eluent used was a 4.5 mmol L^−1^ Na_2_CO_3_ + 1.0 mmol L^−1^ NaHCO_3_ mixture with an addition of 30 vol% ACN. NTf_2_-anion purity could be determined within an error range of up to 0.5% while the IL purity could be determined within an error range of up to 10%.

For the analysis of Pt leaching or Pt residues after catalysis, graphite furnace atomic absorption spectrometry (GF-AAS) was made using a Perkin Elmer PinAAcle 900T (Perkin Elmer LAS GmbH, Rodgau-Jügesheim, Germany) spectrometer. Solutions of 0.050 mg_Pt_ L^−1^, 0.100 mg_Pt_ L^−1^, 0.200 mg_Pt_ L^−1^, and 0.400 mg_Pt_ L^−1^ were prepared from an AAS Pt standard (Fluka, 1000 ± 4 mg L^−1^, 5% HCl) for calibration. The samples contained 0.2 mL of the product solution and were further diluted with ethanol to achieve values within the calibration range of 0.050 to 0.400 mg_Pt_ L^−1^. The obtained values can deviate within a range of ±10%.

Gas chromatography (GC) was performed with a Thermo Finnigan Trace GC Ultra, Column BPX5 (column length: 15 m), combined with the mass spectrometer (MS) Thermo Finnigan Trace DSQ (Thermo Fischer Scientific GmbH, Dreieich, Germany), using the EI ionization method with 70 eV and a source temperature of 200 °C.

### 3.2. Synthesis of Pt-NPs in IL and EG/IL mixtures

*(TAAIL)Pt-NPs*: Pt-NPs in TAAILs were synthesized as described previously [21,22]. In general, MeCpPtMe_3_ and the corresponding IL were placed in a 10 mL microwave vessel. The mass of the Pt precursor was set to achieve 2 wt% of Pt-NPs in IL when assuming quantitative conversion in a batch of about 500 mg IL (~0.4 mL). The dispersion was stirred for at least 6 h and afterward heated in the microwave reactor (200 °C, 40 W, 10 min holding time). To remove the IL several washing steps (with ultrasonication and centrifugation) were performed with 3 mL of ACN per washing step until a clear colorless centrifugate was obtained. The (TAAIL)Pt-NP residue was dried in a high vacuum (5 × 10^−3^ mbar) for 2 h. The yield of Pt-NPs was quantitative.

*(EG/TAAIL)Pt-NP*: Pt-NPs in mixtures of EG and TAAILs were synthesized using a modified version of the surfactant-free polyol process by Quinson et al. [30]. In general, K_2_PtCl_6_, NaOH, EG, and the TAAIL (with 10, 25, 50, and 75 wt% IL in EG/IL) were placed in a 10 mL microwave glass vessel. Then 12 equivalents of NaOH to Pt were added. The amount of Pt precursor was set to achieve 1 wt% of Pt-NPs in EG/TAAIL at quantitative conversion, with batch sizes of about 600 mg EG/TAAIL. The dispersion was stirred for at least 6 h and heated afterward in the microwave reactor (170 °C, 100 W, 10 min holding time). To remove the EG and IL the black dispersion was washed (ultrasonicated and centrifugated) several times with 3 mL of ACN each until a clear colorless centrifugate was obtained. The black solid was then washed twice (with ultrasonication and centrifugation) with MeOH to remove NaOH residues. The remaining black product was dried in a high vacuum (5 × 10^−3^ mbar) for 2 h to give a quantitative yield of Pt-NPs.

### 3.3. Electrochemical Measurements

For all measurements, a conventional three-electrode cell with a glassy carbon rotating disk working electrode (5 mm diameter), a Pt sheet as a counter electrode (1.5 × 1.5 cm^2^), and a silver/silver chloride reference electrode (Ag/AgCl in 3 mol L^−1^ NaCl solution) was used with 0.5 mol L^−1^ H_2_SO_4_ electrolyte solution and an Interface 1010 potentiate by Gamry Instruments.

As electrochemically active material fresh NP inks were prepared similarly to Beermann et al. [86] where 0.2 mg of the NP component was first mixed with 0.8 mg Vulcan XC-72R. Further, 1 mg of this solid was dispersed in 1 mL of a 1/5 (*v*/*v*) isopropanol/water mixture containing 5 µL Nafion™ 1100W 5 wt% and sonicated for at least 30 min. Next, 20 µL of the ink was deposited onto the working electrode and dried at room temperature with a rotation speed of 120 rpm to form a thin film. The resulting platinum loading on the electrode was 20 µg_Pt_ cm^−2^.

All following measurements were completed under a protective gas atmosphere at a rotation speed of 3600 rpm. Before electrochemical measurements were started, the electrolyte solution was purged with N_2_ for 10 min. The catalyst was activated via potential cycling between −0.10 and 0.30 V_Ag/AgCl_ for 30 cycles with a scan rate of 100 mV s^−1^. To determine the activities of the catalysts, linear sweep voltammograms (LSV) were recorded in a potential range between 0.1 and −0.35 V_Ag/AgCl_ with a scan rate of 10 mV s^−1^. The overpotential was determined at a current density of 10 mA cm^−2^. Polarization curves vs Ag/AgCl were corrected by iR compensation and converted to a reversible hydrogen electrode (RHE), according to *E (RHE)* = *E(Ag/AgCl) + E*° *+ 0.059* V·*pH* with *E*° = 0.211 V. Stability tests were conducted via potential scanning between 0.1 and −0.3 V_Ag/AgCl_ for 1000 cycles at 100 mV s^−1^. Chronoamperometry has been performed as an alternative stability test at a controlled voltage of 63 mV for 1 h at room temperature. Due to the parameters of the measurement, given voltages can deviate by up to 1 mV for all measurements.

### 3.4. Hydrosilylation Reactions

*Method 1:* The catalytic reactions were performed as a two-phase system in a microwave reactor using quartz glass vials of 10 mL. A mixture of K_2_PtCl_6_ (~1.3 µmol Pt, see Table S5 for the molar ratio of substrate/Pt), EG, and IL (~0.2 wt% Pt in a 9/1 ratio of EG/IL) was placed in the glass vial and degassed under vacuum. Afterward, 1.37 mL (12.5 mmol) of phenylacetylene and 2.00 mL (12.5 mmol) of triethylsilane were added to the glass vial under an N_2_ atmosphere, followed by a reaction at 110 °C for 15 min under 30 W of microwave irradiation. The upper product phase was syringed off after centrifugation and analyzed by ^1^H NMR, ^13^C NMR, and GC for the different product species and the conversion. The statistical error of the distal/proximal product ratio and substrate conversion determined by signal intensities in ^1^H NMR is about 5% for both determinations. Conversions above 99% result in significantly larger deviations due to the low intensity of the remaining starting material and are only mentioned as >99%. The statistical error of the distal/proximal product ratio determined by signal intensities in GC is roughly up to 10%. To test the stability of the catalyst, the same number of starting materials was added again to the remaining EG/IL phase and the procedure was repeated.

*Method 2:* The catalytic reactions were performed as a one-phase system in the same glass vials as in method 1. (EG/TAAIL)Pt-NP probes (~5.0 µmol Pt, see Table S5 for the molar ratio of substrate/Pt) were placed in the glass vial followed by the addition of 0.55 mL (5.0 mmol) of phenylacetylene and 0.80 mL (5.0 mmol) of triethylsilane. The reaction was carried out as described in method 1 at 110 °C for 15 min but under 200 W microwave irradiation for 5 min. The product solution was syringed off from the solid catalyst after centrifugation and analyzed as described above. The solid catalyst was reused, and the procedures were repeated to test the catalyst’s stability.

*Method 3:* The catalytic reactions, washing, and characterization were carried out in the same manner as described in method 2 but with (EG/TAAIL)Pt-NP and (TAAIL)Pt-NP probes (see Table S5 for the molar ratio of substrate/Pt) at 200 °C for 5 min under 200 W of microwave irradiation.

## 4. Conclusions

Nine tunable aryl alkyl ionic liquids (TAAIL), including TAAILs with an additional phenyl substitution at the imidazole C2 position, were utilized as reaction media and stabilizer for the microwave-assisted synthesis of Pt-nanoparticles (Pt-NPs) from MeCpPtMe_3_. In an ethylene glycol (EG)/TAAILs mixture the precursor K_2_PtCl_6_ was used. Small Pt-NPs were obtained whose calculated crystallite sizes from PXRD with the Scherrer equation of 3 nm to 5 nm correspond to particle sizes observed by TEM. TEM further illustrated that all samples formed large aggregates of the primary NPs.

The (TAAIL)Pt-NPs and (EG/TAAIL)Pt-NPs showed competitive activities in the electrocatalytic hydrogen evolution reaction. In particular, the Pt-NP sample with EG/[Ph_2-Me_ImPhC_4_][NTf_2_] (EG-MPP4) exhibited a very low overpotential of 32 mV at 10 mA cm^−2^, outperforming the reference material Pt/C 20 wt% with 42 mV. The sample EG-MPP4 also had a low Tafel slope of 19 mV dec^−1^.

The (TAAIL)Pt-NP and (EG/TAAIL)Pt-NP probes could function as catalysts for the hydrosilylation of phenylacetylene with triethylsilane with quantitative conversion in a short time of 5 min. In addition, a two-phase system with an EG/TAAIL phase containing the salt K_2_PtCl_6_ also achieved quantitative conversion in 15 min. In all cases, the short reaction time for quantitative conversion resulted from microwave heating while in literature references work significantly higher reaction times of 1 h for two-phase reactions [44,45] and over 2 h for Pt-NP catalysts were needed [69,75,76,77,78,79]. The samples achieved very high TOF values of up to 43,300 h^−1^. The distal hydrosilylation product was preferentially obtained over the proximal one in all reactions, with a ratio of up to 3.5. However, the recycling and reuse of the catalysts could still not be successfully implemented, in part due to an unexpectedly high degree of Pt leaching into the product solution. Finding the right reaction conditions for IL/Pt-NP catalysts to prevent leaching and deactivation is a challenge for future work. Only then can the full design potential of ionic liquids as reaction media in hydrosilylation catalysis be utilized.

## Figures and Tables

**Table 1:** Anion purity, IL purity, and decomposition temperature of TAAILs utilized in this work.

TAAIL	Color	Anion Purity ^1^ (wt%)	IL purity ^2^ (wt%)	T_Dec_^3^ (°C)
[Ph_4-Me_ImC_4_][NTf_2_]	orange	98.4	92	403
[Ph_4-OMe_ImC_4_][NTf_2_]	brown	>99	95	417
[Ph_4-Br_ImC_4_][NTf_2_]	orange	97.6	98	410
[Ph_4-Br_ImC_5_][NTf_2_]	brown	98.4	102	399
[Ph_2,4-F_ImC_4_][NTf_2_]	brown	>99	97	394
[PhImPhC_4_][NTf_2_]	orange	>99	94	414
[Ph_2-Me_ImPhC_4_][NTf_2_]	yellow	98.3	93	414
[Ph_4-OMe_ImPhC_4_][NTf_2_]	brown	>99	102	410
[Ph_4-Br_ImPhC_4_][NTf_2_]	black	97.6	94	413

^1^ The anion purity as [NTf_2_]^−^ wt% based on the sum of the determined anion ([NTf_2_]^−^, F^−^, Cl^−^, Br^−^) contributions (average from a double determination of two samples, that is four measurements). ^2^ The IL purity as wt% is assessed as the average (double determination of two samples) of the IC-determined triflimide concentration ([NTf_2_]^−^_exp_ in mg/L) relative to the theoretical concentration ([NTf_2_]^−^_theo_ in mg/L) based on the mass of IL (m_exp_(IL)) in 100 mL of solution and assuming 100% purity. At very high IL purities, small measurement errors can result in values above 100%. For further information, see Section S2.3. ^3^ Decomposition temperature from thermogravimetric analysis at a heating rate of 5 K min^−1^ under a nitrogen atmosphere.

**Table 2:** Summary of crystallite and particle sizes of (TAAIL)Pt-NPs.

Sample	TAAIL Used in Synthesis	Crystallite Size ^1^ (nm)	Particle Size ^2^ (nm)
pMP4	[Ph_4-Me_ImC_4_][NTf_2_]	4	3.1 ± 0.6
MOP4	[Ph_4-OMe_ImC_4_][NTf_2_]	3	1.8 ± 0.3
BP4	[Ph_4-Br_ImC_4_][NTf_2_]	4	3.2 ± 0.5
BP5	[Ph_4-Br_ImC_5_][NTf_2_]	5	3.3 ± 0.6
DFP4	[Ph_2,4-F_ImC_4_][NTf_2_]	3	2.2 ± 0.6
PP4	[PhImPhC_4_][NTf_2_]	3	2.3 ± 0.4
MPP4	[Ph_2-Me_ImPhC_4_][NTf_2_]	3	2.4 ± 0.4
MOPP4	[Ph_4-OMe_ImPhC_4_][NTf_2_]	3 ± 1	2.9 ± 0.4
BPP4	[Ph_4-Br_ImPhC_4_][NTf_2_]	4	5.0 ± 1.0

^1^ From PXRD, average crystallite size and standard deviation are determined by applying the Scherrer equation to all reflexes observed in the respective sample. ^2^ From TEM, determined from over 200 evaluated particles.

**Table 3:** Summary of crystallite and particle sizes of (EG/TAAIL)Pt-NPs.

Sample Name	TAAIL Used in Synthesis	Crystallite Size ^1^ (nm)	Particle Size ^2^ (nm)
EG-pMP4	[Ph_4-Me_ImC_4_][NTf_2_]	2	/
EG-MOP4	[Ph_4-OMe_ImC_4_][NTf_2_]	4	1.8 ± 0.3
EG-BP4	[Ph_4-Br_ImC_4_][NTf_2_]	2	/
EG-BP5	[Ph_4-Br_ImC_5_][NTf_2_]	3	3.3 ± 0.6
EG-DFP4	[Ph_2,4-F_ImC_4_][NTf_2_]	3	2.2 ± 0.6
EG-PP4	[PhImPhC_4_][NTf_2_]	5	2.3 ± 0.4
EG-MPP4	[Ph_2-Me_ImPhC_4_][NTf_2_]	3	2.4 ± 0.4
EG-MOPP4	[Ph_4-OMe_ImPhC_4_][NTf_2_]	2	2.9 ± 0.4
EG-BPP4	[Ph_4-Br_ImPhC_4_][NTf_2_]	2	5.0 ± 1.0

^1^ From PXRD, average crystallite size and standard deviation are determined by applying the Scherrer equation to all reflexes observed in the respective sample. ^2^ From TEM, determined from over 200 counted particles.

**Table 4:** Summary of electrochemical parameters for (TAAIL)Pt-NPs and (EG/TAAIL)Pt-NPs.

Sample Name	η_10 mA cm_^−2^ after Activation (mV)	Tafel Slope (mV dec^−1^) ^3^	η_10 mA cm_^−2^ after Stability Test (mV)
*(TAAIL)Pt-NPs*			
pMP4	64	24	/
BP4	n.a. ^1^	/	/
DFP4	n.a. ^1^	/	/
PP4	n.a. ^1^	/	/
MPP4	n.a. ^1^	/	/
MOPP4	83	54	/
BPP4	108	81	/
*(EG/TAAIL)Pt-NPs*			
EG-pMP4	n.a. ^1^	/	/
EG-MOP4	123	78	/
EG-BP4	54	27	70
EG-BP5	58	26	/
EG-DFP4	n.a. ^1^	/	/
EG-PP4	74	44	/
EG-MPP4	32	20	n.a. ^1^
EG-MOPP4	79	46	/
EG-BPP4	39	24	n.a. ^1^
*Reference materials*			
Pt/C 20 wt%	42	25	51
Pt_1_/OLC [66]	38	35	38 ^4^
Pt_3_Ni_2_ NWs-S/C [67]	27	/	/
ALD50Pt/NGNs [68]	50 ^2^	29	50

^1^ The sample did not reach a current density of 10 mA cm^−2^_._ ^2^ Overpotential reported at 16 mA cm^−2^. ^3^ Based on the kinetically controlled area at low overpotentials. ^4^ The stability test consisted of 6000 CV cycles instead of 5000.

**Table 5:** Catalytic hydrosilylation of phenylacetylene with triethylsilane ^1^.

Sample Name	Time (h)	Temp. (°C)	Molar Substrate/Pt Ratio ^2^	Conversion (%) ^3^	d/p Ratio ^4^
**Method 1**, (EG/TAAIL)K_2_PtCl_6_					
(EG/[Ph_4-Br_ImC_4_][NTf_2_])K_2_PtCl_6_	0.25	110	9620	96	3.1
(EG/[Ph_4-Br_ImC_5_][NTf_2_])K_2_PtCl_6_	0.25	110	9620	96	2.5
(EG/[PhImPhC_4_][NTf_2_])K_2_PtCl_6_	0.25	110	9940	99	3.0
(EG/[Ph_2-Me_ImPhC_4_][NTf_2_])K_2_PtCl_6_	0.25	110	10,100	>99	3.1
(EG/[Ph_4-Br_ImPhC_4_][NTf_2_])K_2_PtCl_6_	0.25	110	8790	>99	2.2
**Method 2**, (EG/TAAIL)Pt-NPs					
EG-BP4	0.25	110	1050	38	3.6
EG-PP4	0.25	110	980	57	3.1
EG-BPP4	0.25	110	980	75	3.4
**Method 3**					
(EG/TAAIL)Pt-NPs					
EG-BP4	0.083	200	880	>99	1.5
EG-BP5	0.083	200	980	67	2.2
EG-MPP4	0.083	200	950	>99	2.0
EG-BPP4	0.083	200	990	99	1.8
(TAAIL)Pt-NPs					
BP4	0.083	200	1030	82	2.6
BP5	0.083	200	1140	95	2.2
PP4	0.083	200	880	99	1.5
MPP4	0.083	200	990	>99	2.0

^1^ Additional data for all reactions including the distal/proximal ratio determined with GC can be found in Table S5, Section S5. ^2^ Molar ratios of phenylacetylene substrate to Pt content. A molar ratio of triethylsilane to phenylacetylene of 1.0 was chosen for all reactions. ^3^ Conversion determined from the reaction mixture by ^1^H NMR spectroscopy. The highest TOF values achieved for each method in h^−1^ (turnover frequency (mol_product_ mol_Pt_^−1^ h^−1^)) were 43,300 ((EG/[Ph_2-Me_ImPhC_4_][NTf_2_])K_2_PtCl_6_), 2900 (EG-BPP4) and 12,900 (BP5) for method 1 to 3, respectively. ^4^ Molar ratios of distal (d) to proximal (p) product, determined from ^1^H NMR spectra of the reaction mixture. For GC-determined ratios see Table S5 and Section S5.

**Table 6:** Reference reactions for the catalytic hydrosilylation of phenylacetylene with triethylsilane ^1^.

Sample Name	Time (h)	Temp. (°C)	Molar Substrate/Pt Ratio ^2^	Conversion (%) ^3^	d/p Ratio ^4^
[P_44414_][NTf_2_]/Karstedt [44] ^1^	1	110	10,000	>99	-
[S_222_][NTf_2_]/K_2_PtCl_6_ [45] ^1^	1	110	10,000	~88	-
Pt_1_/NaY [75]	24	110	2440	82	0.3
7.0 nm Pt/SBA-15 [76]	6	70	390	6.8	1.8
Pt/C [77]	4.5	70	4880	91	3.3
Pt-NP [78]	1.3	60	200	82	4.9
Pt-NP [78]	24	60	200	94	9.0
Pt-NP [79]	10	rt	1000	98	6.7
C-Pt/ImIP-2BrB [80]	4	80	2000	79	0.8

^1^ Differing from the other reactions, [P_44414_][NTf_2_] and [S_222_][NTf_2_] were carried out as hydrosilylation reactions of 1-octene and 1,1,1,3,5,5,5-heptamethyltrisiloxane with the Karstedt catalyst and K_2_PtCl_6_ as catalyst, respectively. Further experiments and information can be found in refs. [44,45]. ^2^ Molar ratios of phenylacetylene (1-octene) substrate to Pt content. For both substrates, a molar ratio of 1.0 was chosen with the exception of refs. [75,78], where a ratio of 1.2 of phenylacetylene to triethylsilane was utilized. ^3^ Conversion determined from the reaction mixture by ^1^H NMR spectroscopy. ^4^ Molar ratios of distal (d) to proximal (p) product.

## Data Availability

The data presented in this study are available on request from the corresponding author.

## Supplementary Materials

The following supporting information can be downloaded at https://www.mdpi.com/article/10.3390/molecules28010405/s1. Section S1: Sources of chemicals; S2: Synthesis and characterization of TAAILs; S3: Synthesis parameters and analyses of platinum-nanoparticles (Pt-NPs) in TAAILs and ethylene glycol (EG); S4: Additional electrochemical measurements; S5: Hydrosilylation conversion and product analysis. Reference [87] is cited in the supplementary materials.
