# Supplementary material for: Tunable Aryl Alkyl Ionic Liquid Supported Synthesis of Platinum Nanoparticles and Their Catalytic Activity in the Hydrogen Evolution Reaction and in Hydrosilylation
Source: Molecules. 2023 Jan 3;28(1):405. doi: 10.3390/molecules28010405 (PMC9822459; doi:10.3390/molecules28010405)
Supplement: Supplementary file 1 [file molecules-28-00405-s001.zip › molecules-2104500-supplementary.pdf]

Tunable Aryl Alkyl Ionic Liquid Supported Synthesis of Platinum Nanoparticles and their Catalytic Activity in the Hydrogen Evolution Reaction and in Hydrosilylation

Dennis Woitassek [1,†], Till Strothmann [1,†], Harry Biller [2], Swantje Lerch [2], Henning Schmitz [1], Yefan Song [1], Stefan Roitsch [3], Thomas Strassner [2,*] and Christoph Janiak [1,*]

1. Institut für Anorganische Chemie und Strukturchemie, Heinrich-Heine-Universität Düsseldorf, 40204 Düsseldorf, Germany
2. Physikalische Organische Chemie, Technische Universität Dresden, 01062 Dresden, Germany
3. Institut für Physikalische Chemie, Universität zu Köln, 50939 Köln, Germany

* Correspondence: thomas.strassner@tu-dresden.de (T.S.); janiak@uni-duesseldorf.de (C.J.); Tel.: +49-2118112286 (C.J.)

† Both authors contributed equally to this work.

Contents

Section S1 Sources of chemicals

Section S2 Synthesis and characterization of TAAILs

Section S3 Synthesis parameters and analyses of platinum-nanoparticles (Pt-NPs) in TAAILs and ethylene glycol (EG)

Section S4 Additional electrochemical measurements

Section S5 Hydrosilylation conversion and product analysis

S1 Sources of chemicals

Table S1. Sources for starting materials and solvents.

Items	Manufacturer
n-Butyllithium (1.6 mol L^{-1} in hexane)	Acros organics
Methyllithium (1.6 mol L^{-1} in diethyl ether)	Sigma-Aldrich
1,2-Dibromoethane (>98%)	Fluka
Potassium hexachloridoplatinate(IV) (97 %)	BLDpharm
Potassium iodide (USP, BP, Ph. Eur. pure, pharma grade	PanReac Applichem
Methylcyclopentadienyl dimer (95 %)	Acros organics
Acetonitrile (≥ 99.9%)	VWR Chemicals
Chloroform-d (99.8 atom % D)	Sigma-Aldrich
Phenylacetylene (97%)	Carbolution
Triethylsilane (99%)	Carbolution
Ethylene glycol (99.5%)	Acros organics
Sodium hydroxide (>98.8%)	Chemsolute
Propan-2-ol (≥99.8%)	Fisher chemical
Sulfuric acid (0.5 mol L^{-1} in water)	Chemsolute

The TAAILs mentioned in this work have been provided by the working group of Prof. Dr. Strassner. For further characterization and synthesis, see the works of Lerch *et al.* and Biller *et al.* [1,2].

S2 Synthesis and characterization of TAAILs

S2.1 Synthesis procedure and characterization

The synthesis of the TAAILs mentioned in this work follow the procedures of Lerch *et al.* [1] and Biller *et al.* [2]. In short, the synthesis of 1-aryl-3-alkylmidazolium bis(trifluoromethylsulfonyl)imide TAAILs was done in two steps, from the alkylation of aryl imidazoles with bromoalkanes to the ion exchange from bromide to bis(trifluoromethylsulfonyl)imide. The 1-aryl-2-aryl-3-alkylimidazolium bis(trifluoromethylsulfonyl)imide TAAILs have been obtained after four steps, from the nucleophile addition to the benzimidamide, second to the ring-closing reaction of the imidazole-backbone, third to the alkylation of aryl imidazoles with bromoalkanes and fourth to the anion exchange to bis(trifluoromethylsulfonyl)imide.

N-(4-bromophenyl)benzimidamide

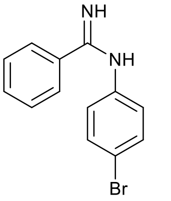

Under an argon atmosphere, sodium hydride (15.0 g, 750 mmol) was dissolved in dry DMSO (200 mL) and cooled externally with an ice bath. 4-Bromoaniline (96.6 g, 550 mmol) and benzonitrile (52.1 g, 500 mmol) were added and the mixture was stirred at 0 °C for 60 minutes and additionally 16 h at room temperature. Ice-water (250 mL) was poured into the mixture under vigorous stirring. The solid was filtered off and washed with *iso*-hexane. The solid was dissolved in an aqueous HCl-solution (1 mol L^{-1}) and filtered. The filtrate was adjusted to pH 11 with an aqueous NaOH solution and extracted with dichloromethane. The combined extracts were washed with water twice, dried with $MgSO_4$ and filtered. After removing the solvent *in vacuo*, the crude product was overlayered with *iso*-hexane and cooled to 0°C for 16 h. The precipitated product was washed with cold *iso*-hexane and dried under high vacuum to yield *N*-(4-bromophenyl)benzimidamide as a beige solid (yield 95.1 g, 346 mmol, 69%).

^{1}H NMR (300 MHz, $CDCl_3$): δ 7.79 (d, *J* = 6.8 Hz, 2H), 7.53 – 7.26 (m, 5H), 6.83 (d, *J* = 8.6 Hz, 2H), 4.95 (s, 2H).

^{13}C NMR (75 MHz, $CDCl_3$): δ 155.6, 148.5, 135.3, 132.6, 130.9, 128.7, 126.9, 123.7, 116.0.

**Elemental analysis** $C_{13}H_{11}BrN_2$	calc.:	C: 56.75%, H: 4.03%, N: 10.18%.
	found:	C: 56.93%, H: 3.99%, N: 10.03%.

1-(4-bromophenyl)-2-phenyl-1H-imidazole

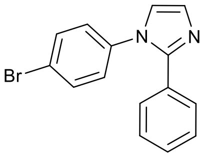

N-(4-bromophenyl)benzimidamide (82.5 g, 30 mmol) and chloroacetaldehyde (56.5 g, 360 mmol, 50 wt% in H_2O) were dissolved in toluene and stirred at 90 °C for 4 h. An NaOH solution was slowly added until a pH of 8 was reached. The reaction was continued for 4 h under reflux. After the solution had cooled down, the mixture was extracted with aqueous HCl (1 mol L^{-1}) twice. The aqueous solution was adjusted with NaOH to pH 8 and extracted with dichloromethane. The organic phase was washed with water and brine and dried with $MgSO_4$. The solvent was concentrated *in vacuo* and poured into an excess of *iso*-hexane. The solution was kept at 0 °C until the product precipitated. The precipitated product was washed with cold *iso*-hexane and dried under high vacuum to yield 1-(4-bromophenyl)-2-phenyl-1*H*-imidazole as a white solid (yield 51.6 g, 173 mmol, 58%).

^{1}H NMR (300 MHz, $CDCl_3$): δ 7.45 (d, *J* = 8.7 Hz, 2H), 7.36 – 7.28 (m, 2H), 7.25 – 7.16 (m, 4H), 7.06 (d, *J* = 1.3 Hz, 1H), 7.02 (d, *J* = 8.7 Hz, 2H).

^{13}C NMR (75 MHz, $CDCl_3$): δ 146.7, 137.5, 132.8, 129.7, 129.1, 128.8, 128.8, 128.5, 127.5, 122.7, 122.1.

**Elemental analysis** $C_{15}H_{11}BrN_2$	calc.:	C: 60.22%, H: 3.71%, N: 9.36%.
	found:	C: 59.91%, H: 3.65%, N: 9.33%.

1-(4-bromophenyl)-3-butyl-2-phenyl-1H-imidazol-3-ium bromide

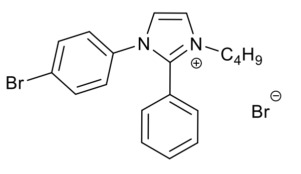

In an Ace pressure tube 1-(4-bromophenyl)-2-phenyl-1*H*-imidazole (12 g, 40 mmol) and 1-bromooctane (6.15 g, 44 mmol) were dissolved in acetonitrile (10 mL). The reaction was stirred for 48 h at 90 °C. When the reaction was complete, the solvent was evaporated and the residue was dissolved in a small amount of dichloromethane (DCM). The solution was then poured into a large excess of diethyl ether. The precipitate which formed was filtered off and washed with diethyl ether. After drying *in vacuo* the product was obtained as a gray solid (yield 16.2 g, 37.1 mmol, 93%).

^{1}H NMR (300 MHz, $CDCl_3$): δ 8.07 (d, *J* = 2.1 Hz, 1H), 7.84 (d, *J* = 2.1 Hz, 1H), 7.66 – 7.57 (m, 2H), 7.56 – 7.31 (m, 7H), 4.17 (t, *J* = 7.6 Hz, 2H), 1.78 (quint, *J* = 7.6 Hz, 2H), 1.21 (sext, *J* = 7.6 Hz, 2H), 0.75 (t, *J* = 7.3 Hz, 3H).

^{13}C NMR (75 MHz, $CDCl_3$): δ 144.33, 133.78, 133.00, 132.44, 131.02, 129.56, 127.85, 124.55, 123.72, 123.18, 121.09, 49.50, 28.28, 21.84, 13.69.

**Elemental analysis** $C_{19}H_{20}Br_2N_2$	calc.:	C: 52.32%, H: 4.62%, N: 6.42%.
	found:	C: 52.07%, H: 4.51%, N: 6.58%.

1-(4-bromophenyl)-3-butyl-2-phenyl-1H-imidazol-3-ium bis(trifluormethanesulfonyl)imid /[Ph$_{4\text{-}Br}$ImPhC$_4$][NTf$_2$]

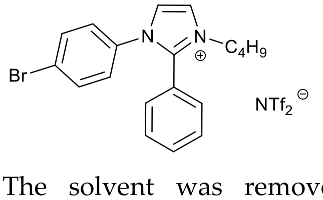

1-(4-Bromophenyl)-3-butyl-2-phenyl-1*H*-imidazol-3-ium bromide (8.72 g, 20 mmol.) was dissolved in DCM (30 mL). $LiNTf_2$ (9.02 g, 22 mmol. 70% aq. solution) and additional water (30 mL) was added to the reaction mixture. The two-phase system was stirred at room temperature for 24 h. The organic phase was extracted twice with DCM, washed twice with water and dried over $MgSO_4$. The solvent was removed *in vacuo* to yield 1-(4-bromophenyl)-3-butyl-2-phenyl-1*H*-imidazol-3-ium bis(trifluormethanesulfonyl)imid (yield 12 g, 18.9 mmol, 94%) as a red liquid.

^{1}H NMR (300 MHz, $CDCl_3$): δ 7.65 – 7.40 (m, 9H), 7.20 (d, *J* = 8.7 Hz, 2H), 4.12 (t, *J* = 7.7 Hz, 2H), 1.81 (quint, *J* = 7.3 Hz, 2H), 1.29 (sext, *J* = 7.6 Hz, 2H), 0.86 (t, *J* = 7.3 Hz, 3H).

^{13}C NMR (75 MHz, $CDCl_3$): δ 144.7, 133.7, 133.4, 132.9, 130.6, 130.0, 127.6, 125.0, 123.6, 122.7, 122.1, 120.8, 49.4, 31.8, 19.6, 13.3.

^{19}F NMR (282 MHz, $CDCl_3$): δ -78.7.

**Elemental analysis** $C_{21}H_{20}Br_2F_6N_2O_4S_2$	calc.:	C: 39.63%, H: 3.17%, N: 6.60%, S: 10.08%.
	found:	C: 39.56%, H: 2.84%, N: 6.93%, S: 10.28%.

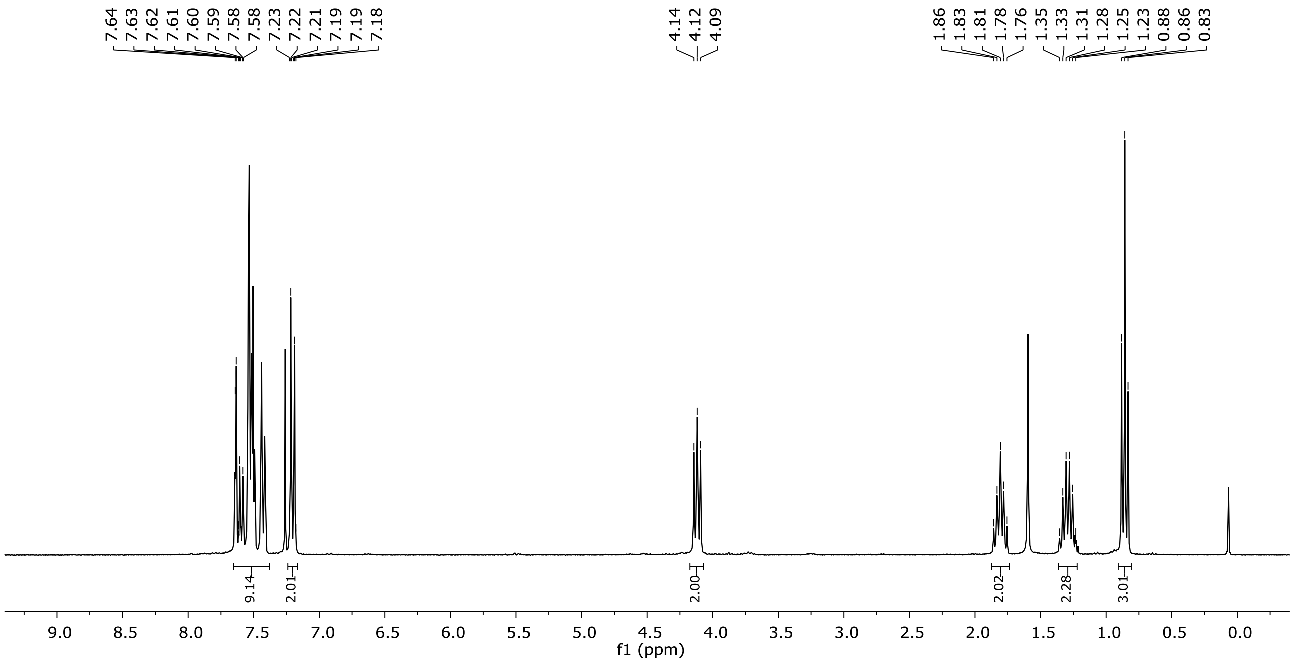

Figure S1. ^{1}H NMR spectrum (300 MHz, $CDCl_3$) of [$Ph_{4\text{-}Br}ImPhC_4$][NTf_2].

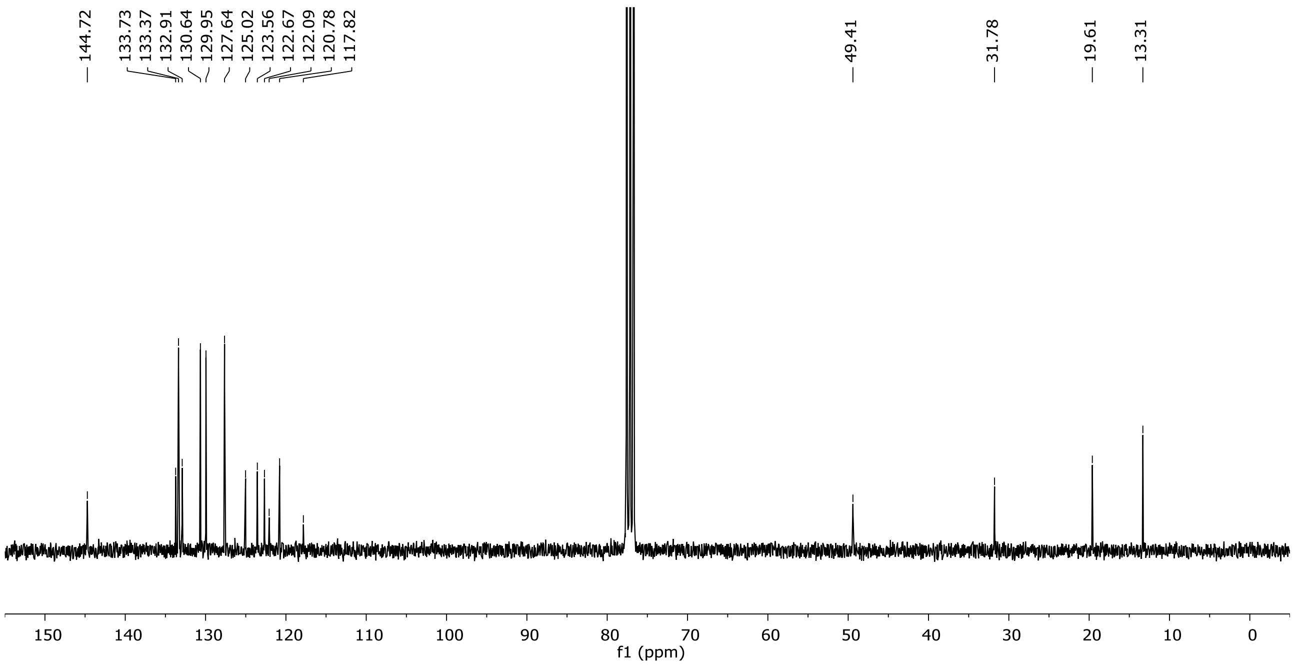

Figure S2. ^{13}C NMR spectrum ($CDCl_3$, 75 MHz) of [$Ph_{4\text{-}Br}ImPhC_4$][NTf_2].

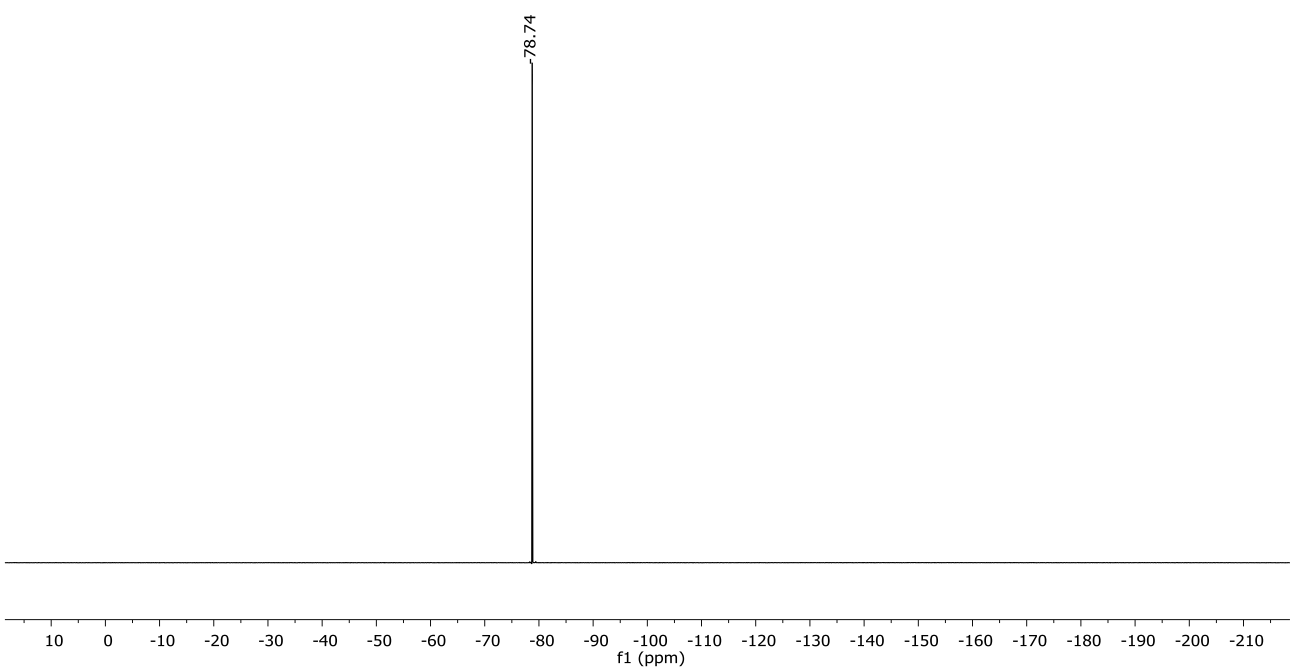

Figure S3. ^{19}F NMR spectrum ($CDCl_3$, 282 MHz) of [$Ph_{4\text{-}Br}ImPhC_4$][NTf_2].

S2.2 Nuclear-magnetic resonance (NMR) spectrometry

[$Ph_{4\text{-}Me}ImC_4$][NTf_2]

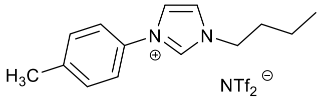

^{1}H NMR (300 MHz, $CDCl_3$): δ 9.04 (s, 1H), 7.60 (t, *J* = 1.9 Hz, 1H), 7.53 (t, *J* = 1.9 Hz, 1H), 7.47 – 7.41 (m, 2H), 7.38 – 7.33 (m, 2H), 4.30 (t, *J* = 7.6 Hz, 2H), 2.42 (s, 3H), 1.98 – 1.84 (m, 2H), 1.41 (sext, *J* = 7.4 Hz, 2H), 0.97 (t, *J* = 7.4 Hz, 3H).

^{13}C NMR (75 MHz, $CDCl_3$): δ 141.3, 134.0, 132.0, 131.2, 123.3, 122.0, 121.8, 119.9 (d, *J* = 321.2 Hz), 50.5, 32.1, 21.2, 19.5, 13.4.

^{19}F NMR (282 MHz, $CDCl_3$): δ -78.9.

[$Ph_{4\text{-}OMe}ImC_4$][NTf_2]

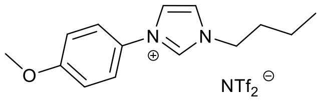

^{1}H NMR (300 MHz, $CDCl_3$): δ 9.01 (s, 1H), 7.57 – 7.44 (m, 4H), 7.10 – 7.01 (m, 2H), 4.30 (t, *J* = 7.5 Hz, 2H), 3.86 (s, 3H), 1.99 - 1.83 (m, 2H), 1.49 – 1.33 (m, 2H), 0.98 (t, *J* = 7.4 Hz, 3H).

^{13}C NMR (75 MHz, $CDCl_3$): δ 161.3, 134.2, 127.3, 123.9, 123.0, 122.0, 119.9 (d, *J* = 321.2 Hz), 115.7, 55.9, 50.5, 32.1, 19.6, 13.4.

^{19}F NMR (282 MHz, $CDCl_3$): δ -78.9.

[Ph$_{4\text{-Br}}$ImC$_4$][NTf$_2$]

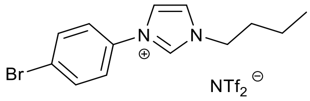

^{1}H NMR (300 MHz, $CDCl_3$): δ 9.09 (s, 1H), 7.73 – 7.64 (m, 3H), 7.56 – 7.44 (m, 3H), 7.20 (d, *J* = 8.7 Hz, 2H), 4.29 (t, *J* = 7.6 Hz, 2H), 1.98 – 1.85 (m, 2H), 1.41 (sext, *J* = 7.4 Hz, 2H), 0.97 (t, *J* = 7.4 Hz, 3H).

^{13}C NMR (75 MHz, $CDCl_3$): δ 134.2, 133.9, 133.4, 124.9, 123.9, 123.6, 121.8, 119.9 (d, *J* = 321.2 Hz), 50.7, 32.0, 19.5, 13.4.

^{19}F NMR (282 MHz, $CDCl_3$): δ -78.9.

[Ph$_{4\text{-Br}}$ImC$_5$][NTf$_2$]

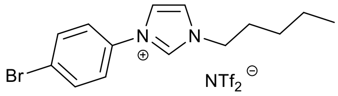

^{1}H NMR (300 MHz, $CDCl_3$): δ 9.16 (s, 1H), 7.74 – 7.62 (m, 3H), 7.54 – 7.47 (m, 3H), 4.30 (t, *J* = 7.6 Hz, 2H), 1.94 (quint, *J* = 7.5 Hz, 2H), 1.40 – 1.32 (m, 4H), 0.94 – 0.87 (m, 3H).

^{13}C NMR (75 MHz, $CDCl_3$): δ 134.4, 133.9, 133.4, 125.0, 123.9, 123.4, 121.7, 119.9 (d, *J* = 321.2 Hz), 51.0, 29.8, 28.3, 22.1, 13.8.

^{19}F NMR (282 MHz, $CDCl_3$): δ -78.9.

[Ph$_{2,4\text{-F}}$ImC$_4$][NTf$_2$]

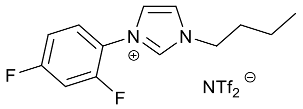

^{1}H NMR (300 MHz, $CDCl_3$): δ 9.04 (s, 1H), 7.77 – 7.66 (m, 1H), 7.58 – 7.52 (m, 2H), 7.18 – 7.05 (m, 2H), 4.33 (t, *J* = 7.6 Hz, 2H), 2.01 – 1.88 (m, 2H), 1.42 (dq, *J* = 14.7 Hz, 7.4 Hz, 2H), 0.99 (t, *J* = 7.3 Hz, 3H).

^{13}C NMR (75 MHz, $CDCl_3$): δ 163.9 (dd, *J* = 256.2 Hz, 11.1 Hz), 155.6 (dd, *J* = 255.9 Hz, 12.6 Hz), 136.2, 127.8 (d, *J* = 10.4 Hz), 123.7, 123.0, 119.8 (d, *J* = 321.2 Hz), 118.9 (dd, *J* = 11.6 Hz, 4.2 Hz), 113.6 (dd, *J* = 23.1 Hz, 3.9 Hz), 106.1 (dd, *J* = 27.1 Hz, 22.7 Hz), 50.8, 31.9, 19.5, 13.4.

^{19}F NMR (282 MHz, $CDCl_3$): δ -79.0, -103.0 (d, *J* = 8.7 Hz), -119.4 (d, *J* = 8.7 Hz).

[PhImPhC$_4$][NTf$_2$]

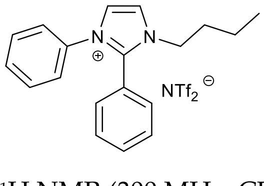

^{1}H NMR (300 MHz, $CDCl_3$): δ 7.67 (d, *J* = 2.2 Hz, 1H), 7.61 – 7.34 (m, 9H), 7.31 – 7.25 (m, 2H), 4.14 (t, *J* = 7.6 Hz, 2H), 1.81 (quint, *J* = 7.6 Hz, 2H), 1.29 (sext, *J* = 7.1 Hz, 2H), 0.86 (t, *J* = 7.3 Hz, 3H).

^{13}C NMR (75 MHz, $CDCl_3$): δ 144.6, 132.7, 130.6, 130.2, 129.8, 126.0, 123.6, 122.6, 121.0, 120.0 (d, *J* = 321.6 Hz), 49.3, 31.8, 19.6, 13.3.

^{19}F NMR (282 MHz, $CDCl_3$): δ -78.8.

[Ph$_{2\text{-Me}}$ImPhC$_4$][NTf$_2$]

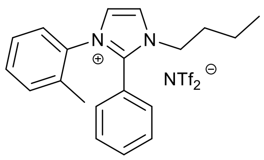

^{1}H NMR (300 MHz, CDCl$_3$): δ 7.76 (d, *J* = 2.1 Hz, 1H), 7.59 – 7.21 (m, 10H), 4.20 (t, *J* = 7.6 Hz, 2H), 2.05 (s, 3H), 1.84 (quint, *J* = 7.6 Hz, 2H), 1.30 (dq, *J* = 14.5 Hz, 7.3 Hz, 2H), 0.87 (t, *J* = 7.3 Hz, 3H).

^{13}C NMR (75 MHz, CDCl$_3$): δ 145.1, 134.2, 133.6, 132.8, 131.7, 131.3, 130.2, 129.8, 127.9, 127.8, 123.7, 122.9, 120.9, 120.0 (d, *J* = 321.5 Hz), 49.5, 31.9, 19.6, 17.4, 13.4.

^{19}F NMR (282 MHz, CDCl$_3$): δ -78.8.

[Ph$_{4\text{-OMe}}$ImPhC$_4$][NTf$_2$]

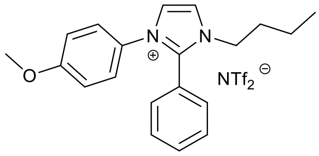

^{1}H NMR (300 MHz, CDCl$_3$): δ 7.64 – 7.38 (m, 7H), 7.23 – 7.16 (m, 2H), 6.89 – 6.82 (m, 2H), 4.11 (t, *J* = 7.6 Hz, 2H), 3.77 (s, 3H), 1.80 (quint, *J* = 7.6 Hz, 2H), 1.29 (sext, *J* = 7.3 Hz, 2H), 0.85 (t, *J* = 7.3 Hz, 3H).

^{13}C NMR (75 MHz, CDCl$_3$): δ 160.9, 144.7, 132.6, 130.6, 129.8, 127.3, 127.3, 123.8, 122.3, 121.1, 120.0 (d, *J* = 321.7 Hz), 115.2, 55.7, 49.5, 31.9, 19.6, 13.3.

^{19}F NMR (282 MHz, CDCl$_3$): δ -78.7.

[Ph$_{4\text{-Br}}$ImPhC$_4$][NTf$_2$]

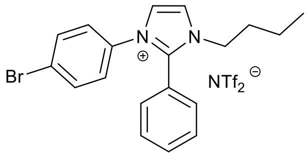

Peak-positions are found in the section 2.1 above.

S2.3 Ion chromatography (IC)

Calculated with Equation S1 and S2, the TAAIL purity is assessed as the average IC-determined triflimide concentration ($[NTf_2]^-_{exp}$ in mg/L, averaged from a double determination of two samples; thus four measurements) relative to the theoretical concentration ($[NTf_2]^-_{theo}$ in mg L^{-1}) based on the sample preparation by dissolving a defined mass of IL ($m_{exp}(IL)$) in 100 mL of eluent (V). M(anion) and M(IL) are the molar mass of the $[NTf_2]^-$-anion and IL, respectively.

$$[NTf_2]^-_{theo} = m_{exp}(IL) \times M(anion) / M(IL) / V \qquad \text{(Eq. S1)}$$

$$\text{IL purity [\%]} = [NTf_2]^-_{exp} / [NTf_2]^-_{theo} \times 100\% \qquad \text{(Eq. S2)}$$

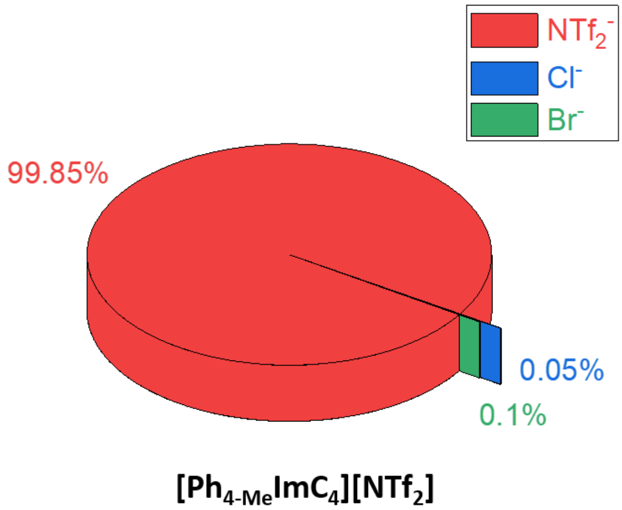

Figure S4. Anion weight-percentages obtained by IC of $[Ph_{4\text{-}Me}ImC_4][NTf_2]$.

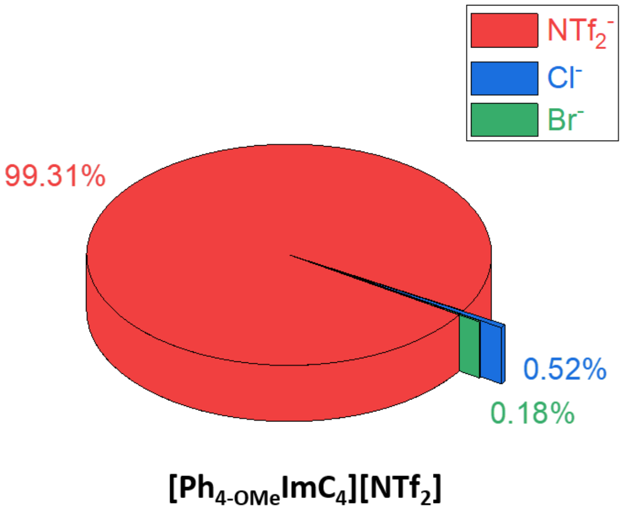

Figure S5. Anion weight-percentages obtained by IC measurements of $[Ph_{4\text{-}OMe}ImC_4][NTf_2]$.

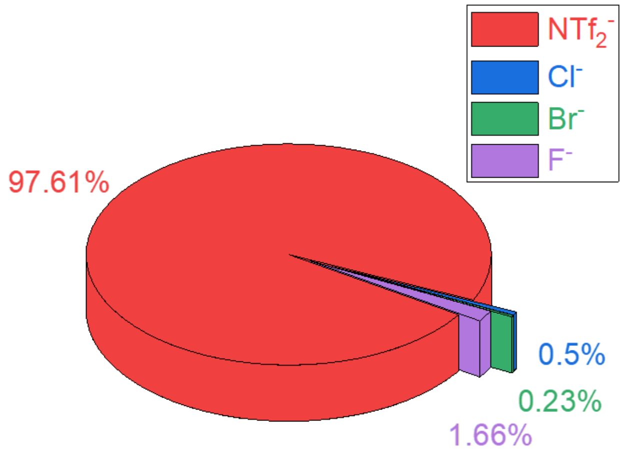

[$Ph_{4\text{-}Br}ImC_4$][NTf_2]

Figure S6. Anion weight-percentages obtained by IC measurements of [$Ph_{4\text{-}Br}ImC_4$][NTf_2].

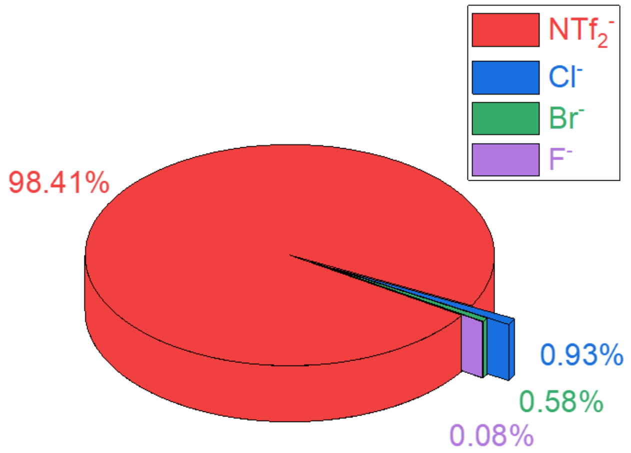

[$Ph_{4\text{-}Br}ImC_5$][NTf_2]

Figure S7. Anion weight-percentages obtained by IC measurements of [$Ph_{4\text{-}Br}ImC_5$][NTf_2].

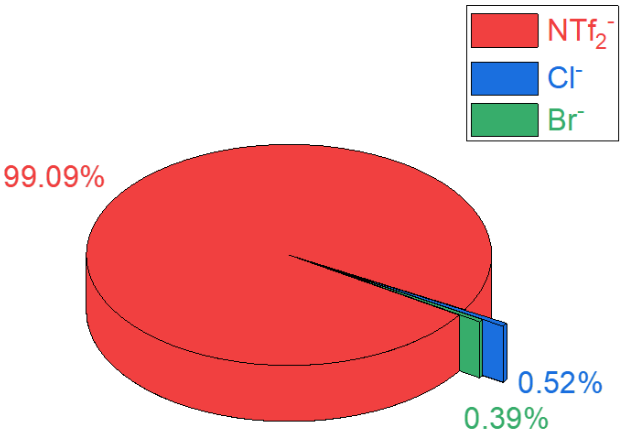

[$Ph_{2,4\text{-}F}ImC_4$][NTf_2]

Figure S8. Anion weight-percentages obtained by IC measurements of [$Ph_{2,4\text{-}F}ImC_4$][NTf_2].

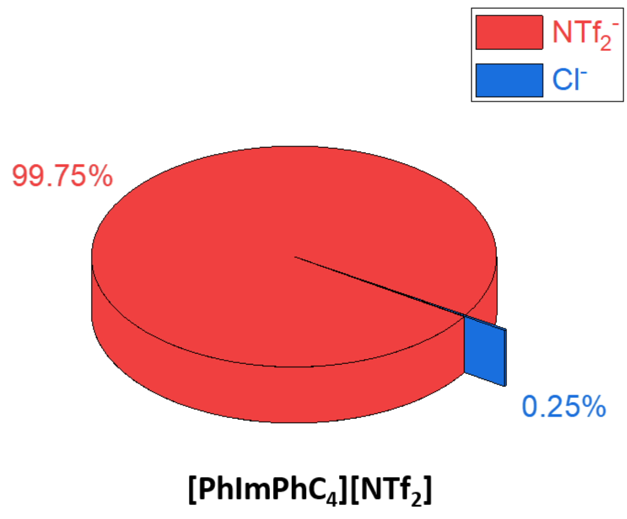

Figure S9 Anion weight-percentages obtained by IC measurements of [PhImPhC$_4$][NTf$_2$].

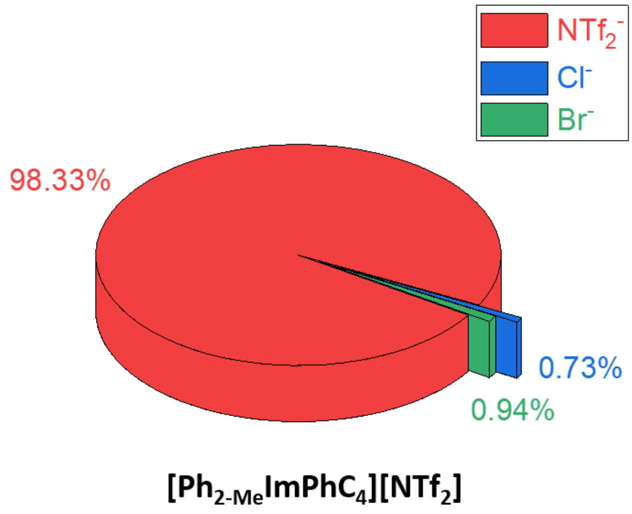

Figure S10. Anion weight-percentages obtained by IC measurements of [Ph$_{2\text{-Me}}$ImPhC$_4$][NTf$_2$].

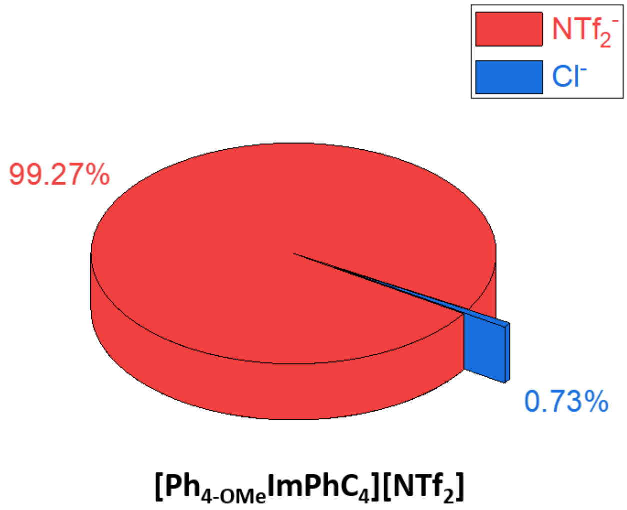

Figure S11. Anion weight-percentages obtained by IC measurements of [Ph$_{4\text{-OMe}}$ImPhC$_4$][NTf$_2$].

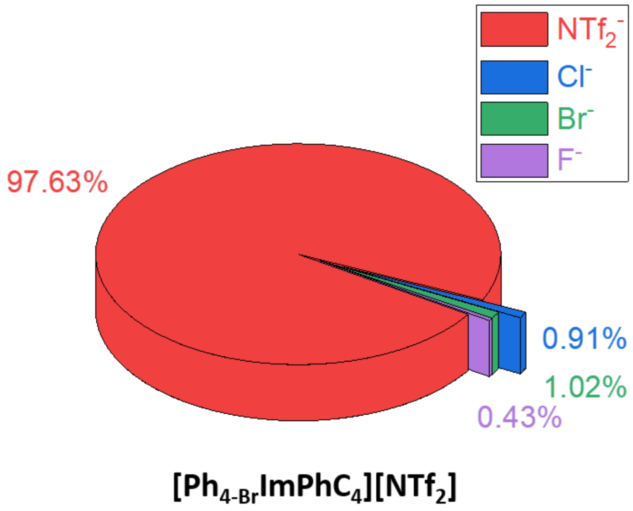

Figure S12. Anion weight-percentages obtained by IC measurements of [$Ph_{4\text{-}Br}ImPhC_4$][NTf_2].

Table S2. Summary of the TAAIL anion composition in weight percentages (wt%) from IC.

TAAIL	NTf_2^- (wt%)	Cl^- (wt%)	Br^- (wt%)	F^- (wt%)
[$Ph_{4\text{-}Me}ImC_4$][NTf_2]	99.85	0.05	0.10	-
[$Ph_{4\text{-}OMe}ImC_4$][NTf_2]	99.31	0.52	0.18	-
[$Ph_{4\text{-}Br}ImC_4$][NTf_2]	97.61	0.50	0.23	1.66
[$Ph_{4\text{-}Br}ImC_5$][NTf_2]	98.41	0.93	0.58	0.08
[$Ph_{2,4\text{-}F}ImC_4$][NTf_2]	99.09	0.52	0.39	-
[$PhImPhC_4$][NTf_2]	99.75	0.25	-	-
[$Ph_{2\text{-}Me}ImPhC_4$][NTf_2]	98.33	0.73	0.94	-
[$Ph_{4\text{-}OMe}ImPhC_4$][NTf_2]	99.27	0.73	-	-
[$Ph_{4\text{-}Br}ImPhC_4$][NTf_2]	97.63	0.91	1.02	0.43

S2.4 Thermogravimetric analysis (TGA)

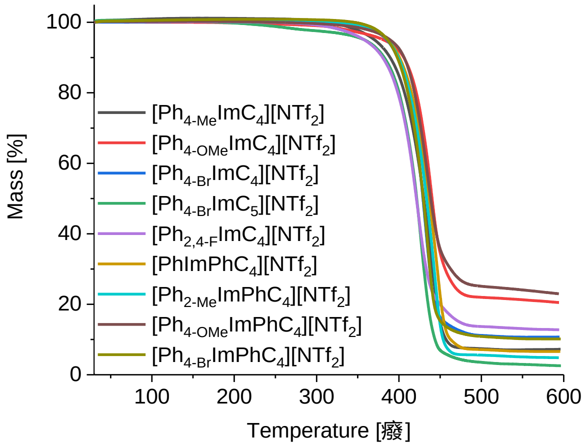

Figure S13. TGA curves of ILs between 30-600 °C at 5 K min^{-1} in N_2 atmosphere.

S3 Synthesis parameters and analyses of platinum-nanoparticles (Pt-NPs) in TAAILs and ethylene glycol (EG)

S3.1 Analyses overview

Table S3. Pt-NPs synthesized in TAAILs ((TAAIL)Pt-NPs) with the corresponding wt% Pt, crystallite size and particle size.

Sample name	TAAIL	Pt wt% [1]	Average crystallite size [2] (nm)	Average particle size [3] (nm)
pMP4	$[Ph_{4\text{-}Me}ImC_4][NTf_2]$	2	4	3.1 ± 0.6
MOP4	$[Ph_{4\text{-}OMe}ImC_4][NTf_2]$	2	3	1.8 ± 0.3
BP4	$[Ph_{4\text{-}Br}ImC_4][NTf_2]$	2	4	3.2 ± 0.5
BP5	$[Ph_{4\text{-}Br}ImC_5][NTf_2]$	2	5	3.3 ± 0.6
DFP4	$[Ph_{2,4\text{-}F}ImC_4][NTf_2]$	2	3	2.2 ± 0.6
PP4-1wt	$[PhImPhC_4][NTf_2]$	1	3	/
PP4	$[PhImPhC_4][NTf_2]$	2	3	2.3 ± 0.4
MPP4-1wt	$[Ph_{2\text{-}Me}ImPhC_4][NTf_2]$	1	3	/
MPP4	$[Ph_{2\text{-}Me}ImPhC_4][NTf_2]$	2	3	2.4 ± 0.4
MOPP4-1wt	$[Ph_{4\text{-}OMe}ImPhC_4][NTf_2]$	1	3	/
MOPP4	$[Ph_{4\text{-}OMe}ImPhC_4][NTf_2]$	2	3 ± 1	2.9 ± 0.4
BPP4-1wt	$[Ph_{4\text{-}Br}ImPhC_4][NTf_2]$	1	4	/
BPP4	$[Ph_{4\text{-}Br}ImPhC_4][NTf_2]$	2	4	5.0 ± 1.0

[1] Pt-NPs were also synthesized with 1 wt% Pt in TAAIL dispersion for comparison. [2] Average crystallite size and standard deviation were determined by applying the Scherrer equation (1) to the strongest reflexes ((111), (200), (220), (311)) observed in the powder X-ray diffraction (PXRD) pattern:

$$L = K \times \lambda/(\Delta(2\theta) \times \cos\theta) \quad \text{(Eq. S3)}$$

with L as average crystallite size, K as dimensionless shape factor (here 1), λ as wavelength (in nm), $\Delta(2\theta)$ as full width at half maximum (FWHM) in radians and θ as Bragg angle (in °). [3] Determined from at least 200 counted particles in TEM images.

Table S4. Crystallite size and particle size of Pt-NP synthesized in mixtures of ethylene glycol (EG) and TAAILs ((EG/TAAIL)Pt-NPs) with 1 wt% Pt.[1]

Sample name[1]	Utilized TAAIL	EG:IL-Ratio	Average crystallite size [2] (nm)	Average particle size [3] (nm)
EG-M4	$[BMIm][NTf_2]$	9/1	2	/
EG-M4-25%	$[BMIm][NTf_2]$	3/1	3	/
EG-M4-50%	$[BMIm][NTf_2]$	1/1	4	/
EG-M4-75%	$[BMIm][NTf_2]$	1/3	7	/
EG-pMP4	$[Ph_{4\text{-}Me}ImC_4][NTf_2]$	9/1	2	1.7 ± 0.3
EG-MOP4	$[Ph_{4\text{-}OMe}ImC_4][NTf_2]$	9/1	4	1.8 ± 0.5
EG-MOP4-50%	$[Ph_{4\text{-}OMe}ImC_4][NTf_2]$	1/1	8	/
EG-MOP4-75%	$[Ph_{4\text{-}OMe}ImC_4][NTf_2]$	1/3	12	/
EG-BP4	$[Ph_{4\text{-}Br}ImC_4][NTf_2]$	9/1	2	1.8 ± 0.3
EG-BP5	$[Ph_{4\text{-}Br}ImC_5][NTf_2]$	9/1	3	2.2 ± 0.4
EG-DFP4	$[Ph_{2,4\text{-}F}ImC_4][NTf_2]$	9/1	3	2.3 ± 0.4
EG-PP4	$[PhImPhC_4][NTf_2]$	9/1	5	2.0 ± 0.5
EG-MPP4	$[Ph_{2\text{-}Me}ImPhC_4][NTf_2]$	9/1	3	/
EG-MOPP4	$[Ph_{4\text{-}OMe}ImPhC_4][NTf_2]$	9/1	2	3.2 ± 0.6
EG-MOPP4-50%	$[Ph_{4\text{-}OMe}ImPhC_4][NTf_2]$	1/1	3	/
EG-MOPP4-75%	$[Ph_{4\text{-}OMe}ImPhC_4][NTf_2]$	1/3	6	/

EG-BPP4	$[Ph_{4-Br}ImPhC_4][NTf_2]$	9/1	2	2.2 ± 0.6
EG-BPP4-50%	$[Ph_{4-Br}ImPhC_4][NTf_2]$	1/1	3	/
EG-BBP4-75%	$[Ph_{4-Br}ImPhC_4][NTf_2]$	1/3	3	/

[1] Pt-NPs were prepared as shown in the main text. In addition, Pt-NP were synthesized with 25 wt%, 50 wt% and 75 wt% of TAAIL in EG. [2] Average crystallite size and standard deviation are determined by applying the Scherrer equation (Eq. S3) to the strongest reflexes ((111), (200), (220), (311)) observed in the PXRD pattern. [3] Determined from at least 200 counted particles in TEM images.

S3.2 Powder X-ray diffractometry (PXRD)

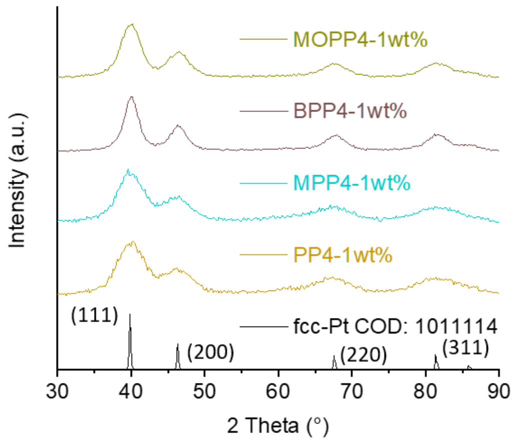

Figure S14. PXRD patterns of (TAAIL)Pt-NPs with 1 wt% Pt in IL. The Pt metal reflections (111), (200), (220) and (311) match to the simulation for fcc-Pt and its indexed reflections (Crystallographic open database fcc-Pt: 1011114). Samples synthesized with 1 wt% Pt in IL do not show differences in crystallinity compared to samples with 2 wt% Pt in IL and are thus not further analysed. PXRD patterns of Pt-NP obtained in 2 wt% Pt in IL are presented in the main text.

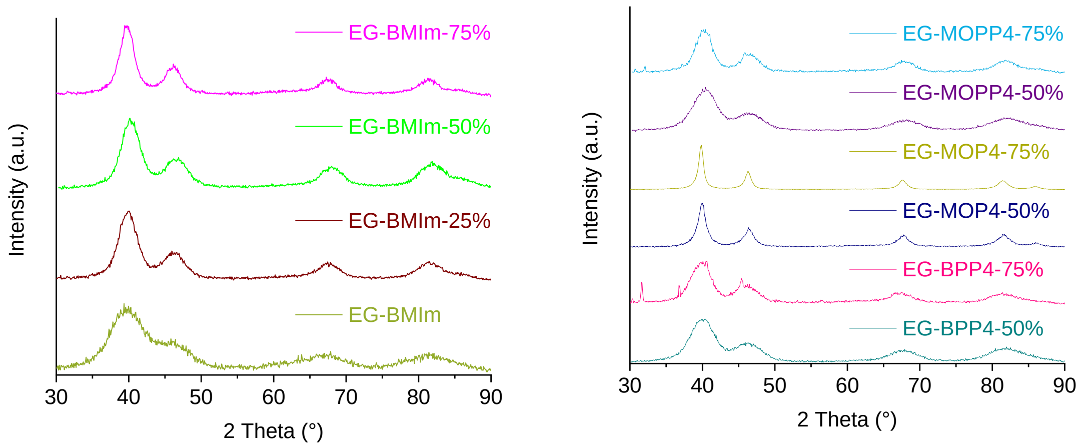

Figure S15. PXRD patterns of (EG/TAAIL)Pt-NP with various EG-IL ratios. The Pt metal reflections (111), (200), (220) and (311) match to the simulation for fcc-Pt and its indexed reflections (Crystallographic open database fcc-Pt: 1011114, cf. simulation, shown in Figure 1 in the main text). With higher amounts of IL the crystallite size increases and are not further analysed.

S3.3 Transmission electron microscopy (TEM)

Images of (TAAIL)Pt-NPs

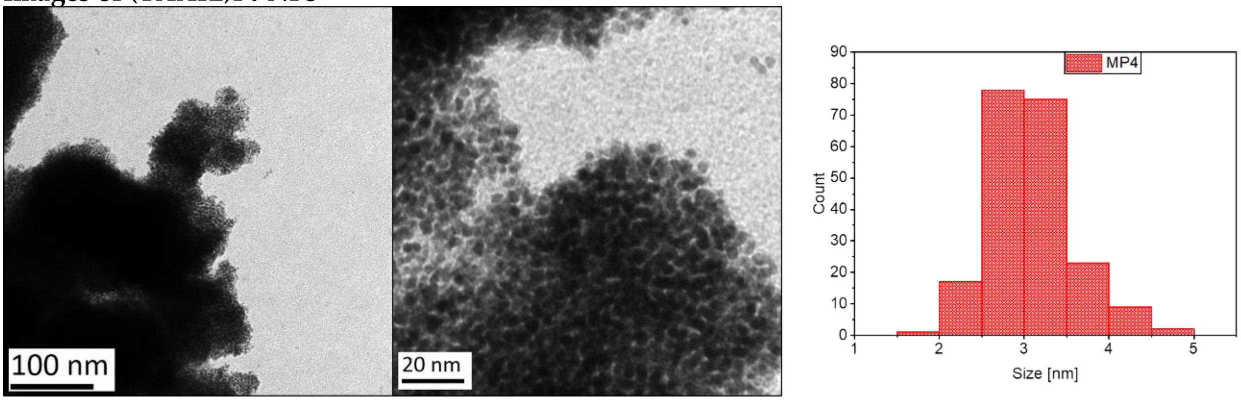

Figure S16. TEM-images and particle-size histogram of sample pMP4. At least 200 particles were analyzed.

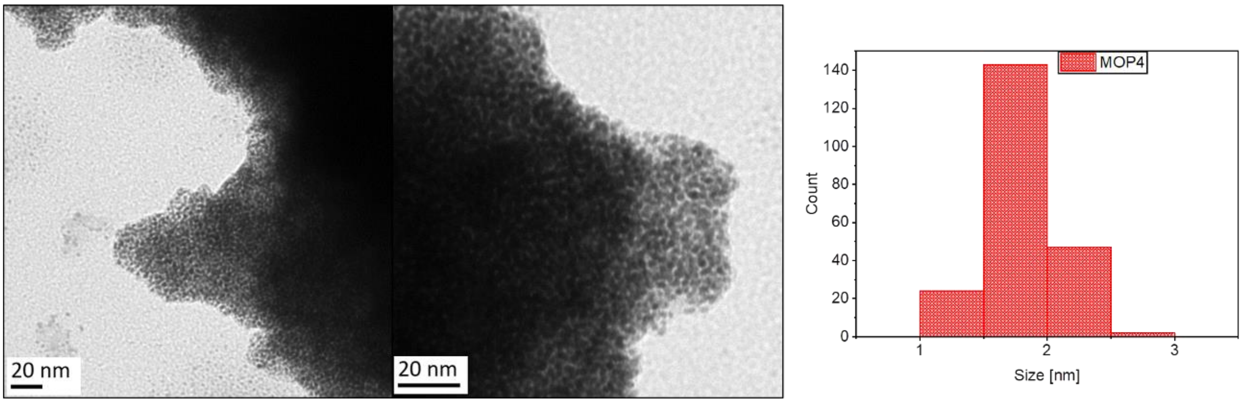

Figure S17. TEM-images and particle-size histogram of sample MOP4. At least 200 particles were analyzed.

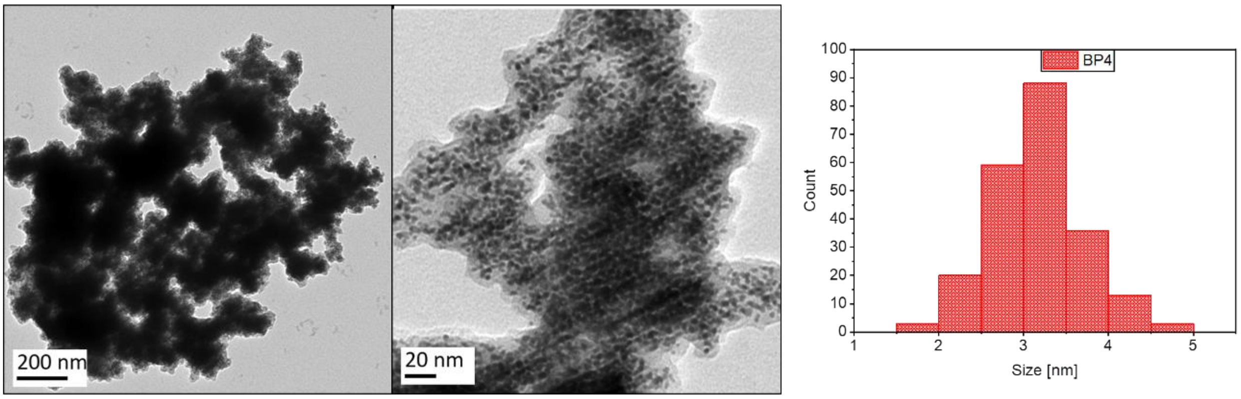

Figure S18. TEM-images and particle-size histogram of sample BP4. At least 200 particles were analyzed.

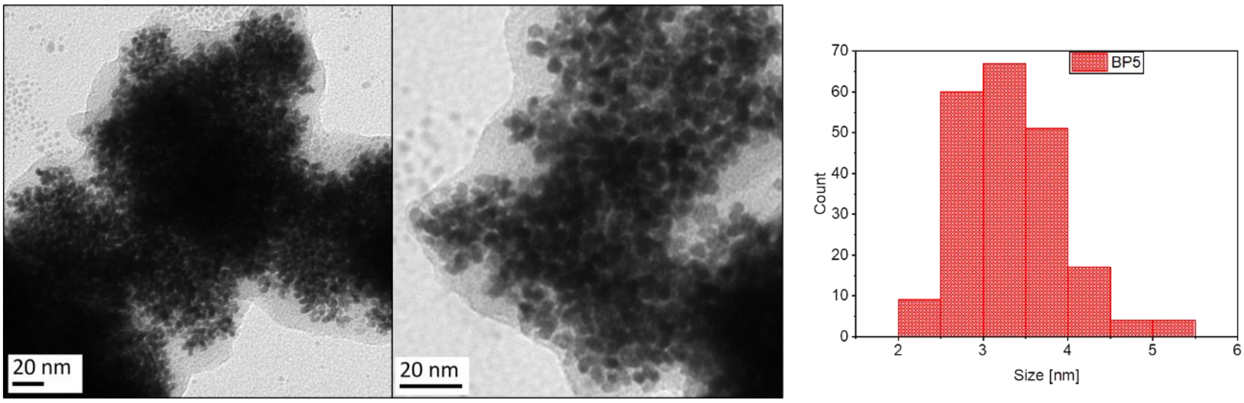

Figure S19. TEM-images and particle-size histogram of sample BP5. At least 200 particles were analyzed.

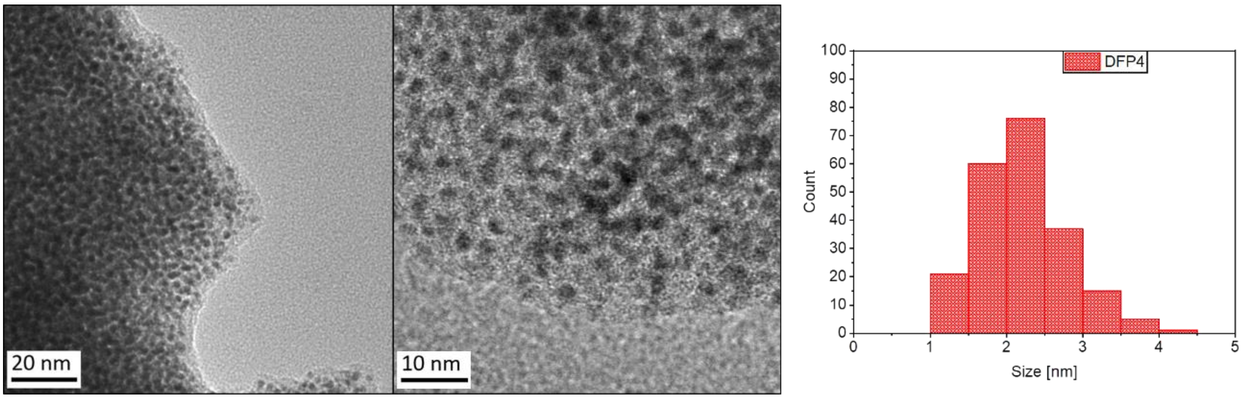

Figure S20. TEM-images and particle-size histogram of sample DFP4. At least 200 particles were analyzed.

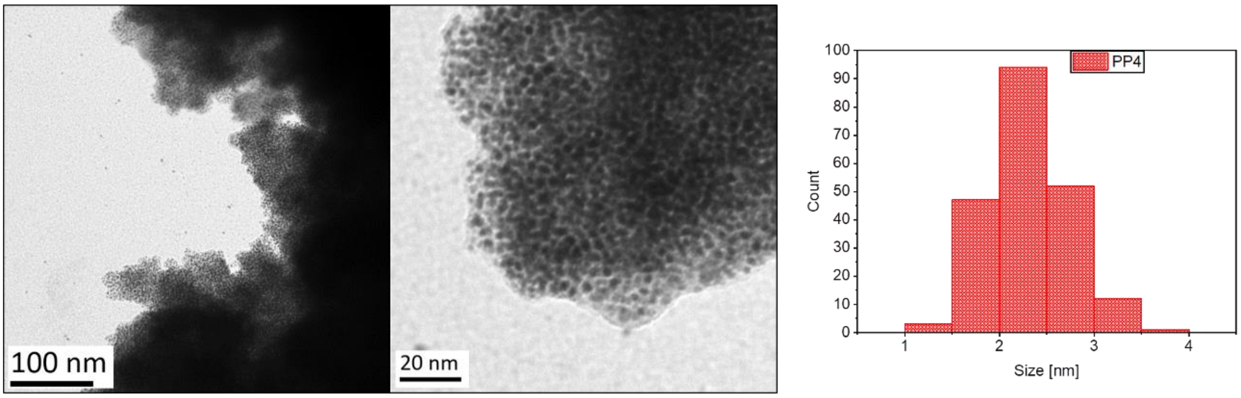

Figure S21. TEM-images and particle-size histogram of sample PP4. At least 200 particles were analyzed.

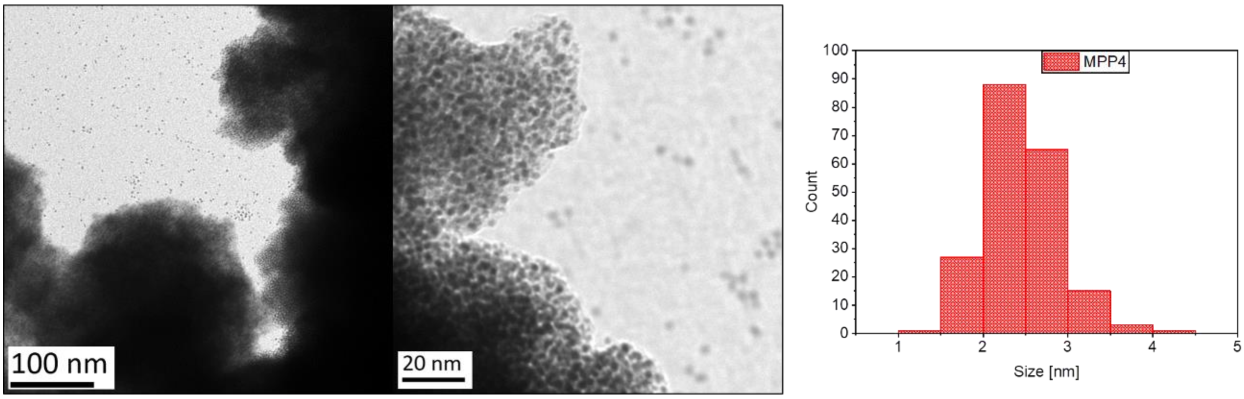

Figure S22. TEM-images and particle-size histogram of sample MPP4. At least 200 particles were analyzed.

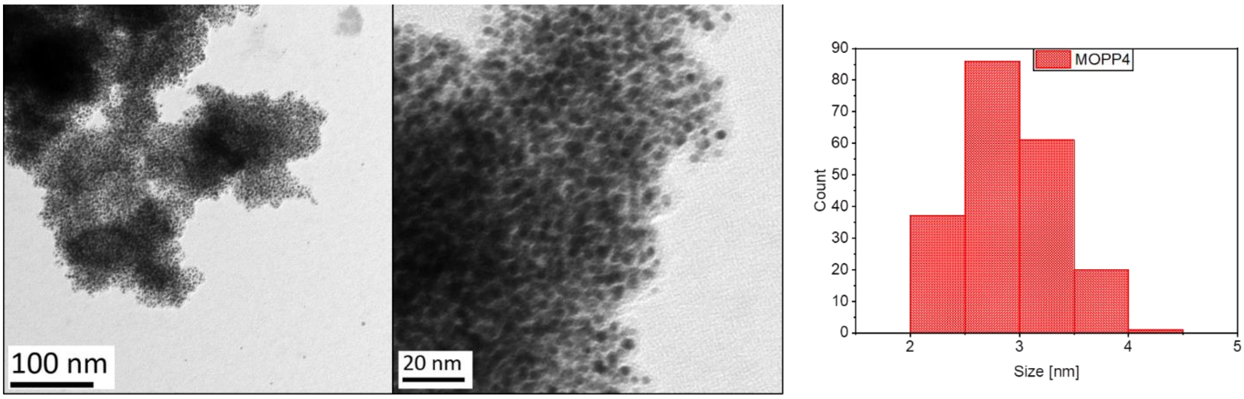

Figure S23. TEM-images and particle-size histogram of sample MOPP4. At least 200 particles were analyzed.

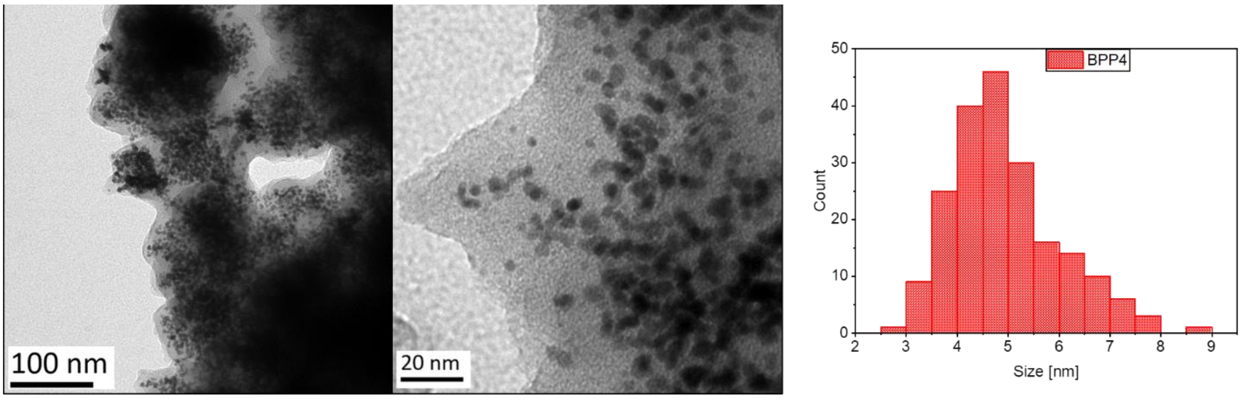

Figure S24. TEM-images and particle-size histogram of sample BPP4. At least 200 particles were analyzed.

Images of (EG/TAAIL)Pt-NPs

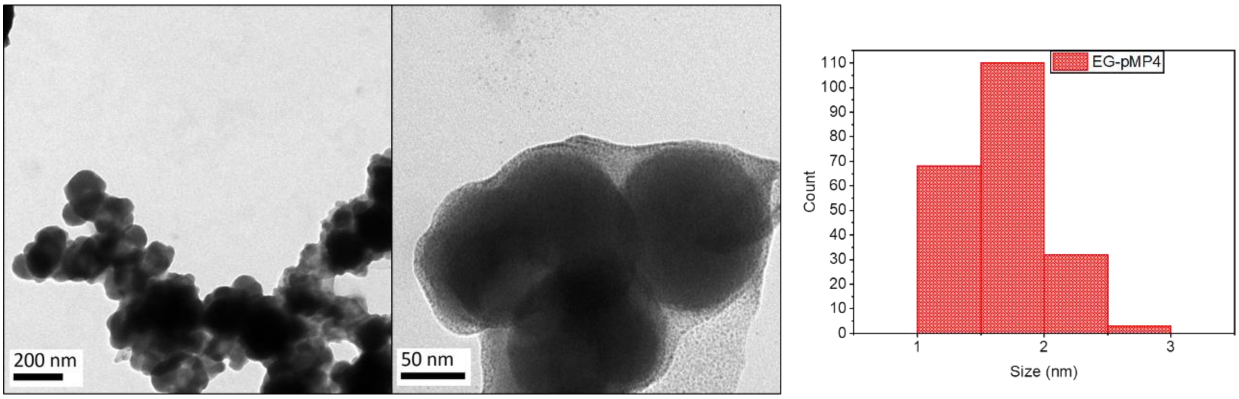

Figure S25. TEM-images and particle-size histogram of sample EG-pMP4. At least 200 particles were analyzed.

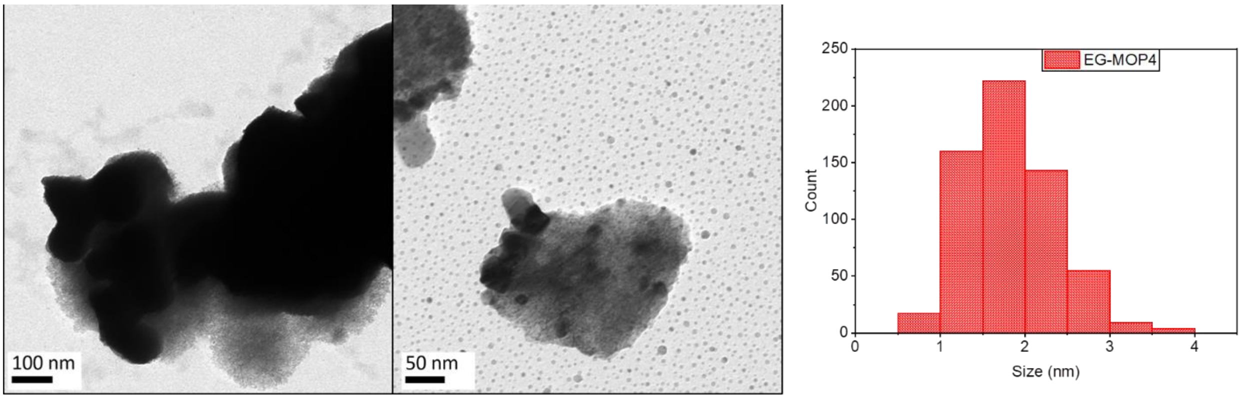

Figure S26. TEM-images and particle-size histogram of sample EG-MOP4. At least 200 particles were analyzed.

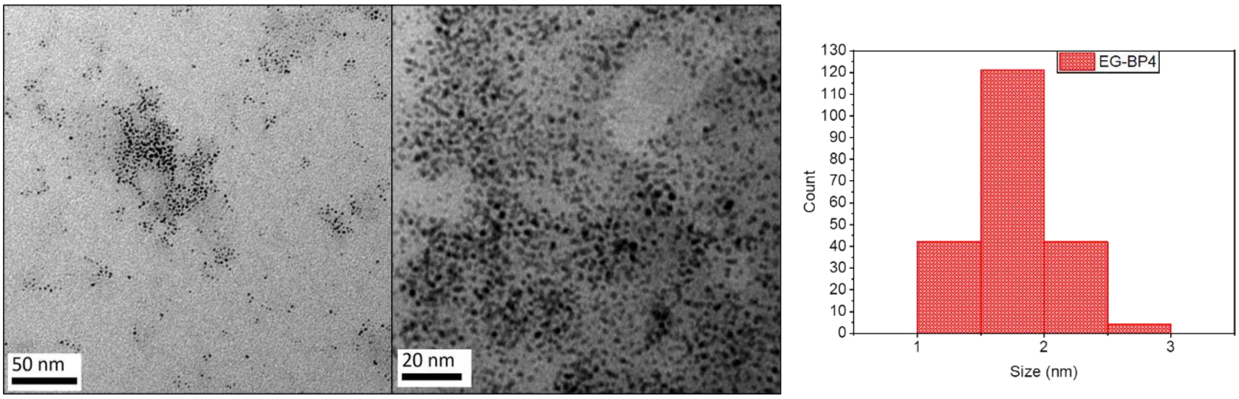

Figure S27. TEM-images and particle-size histogram of sample EG-BP4. At least 200 particles were analyzed.

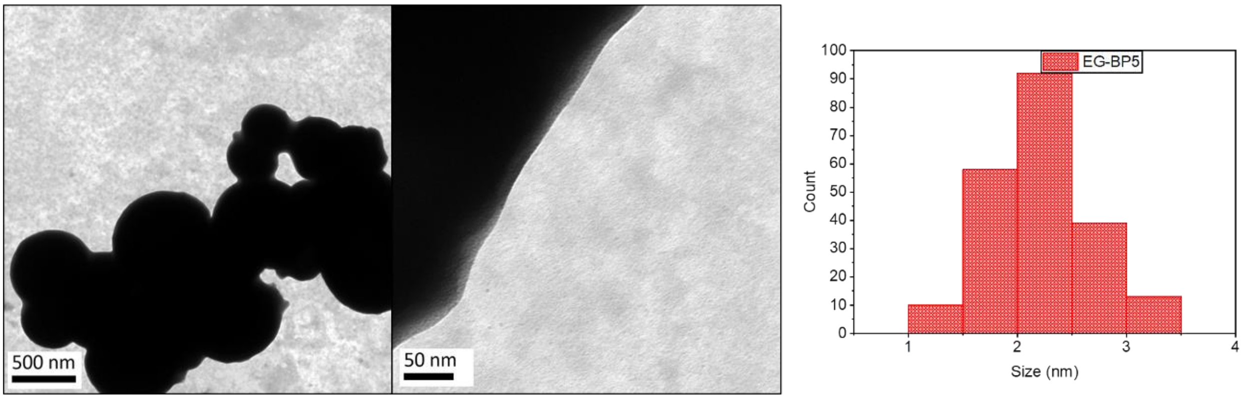

Figure S28. TEM-images and particle-size histogram of sample EG-BP5. At least 200 particles were analyzed.

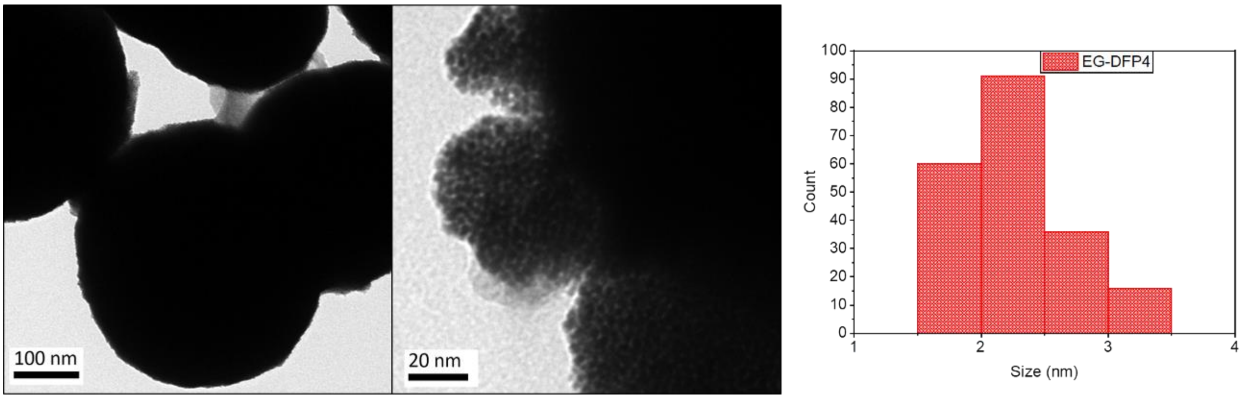

Figure S29. TEM-images and particle-size histogram of sample EG-DFP4. At least 200 particles were analyzed.

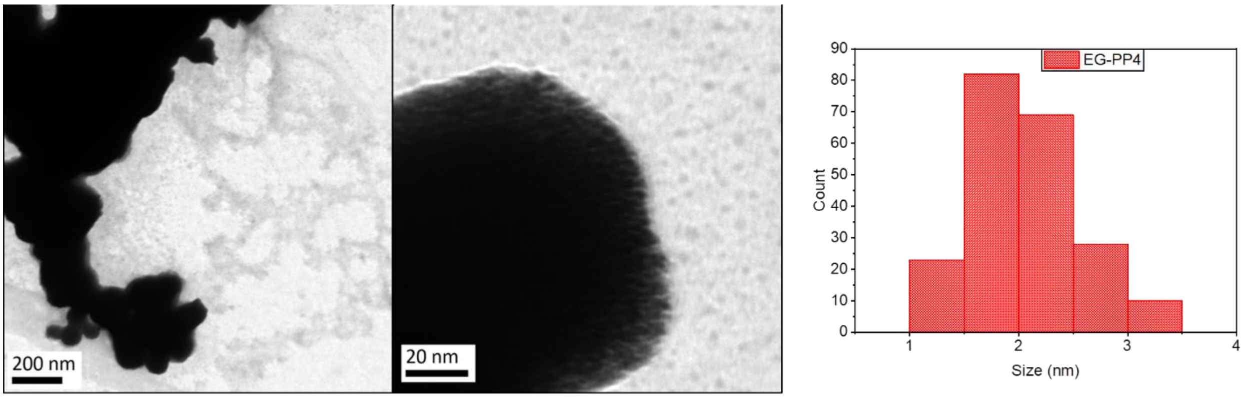

Figure S30. TEM-images and particle-size histogram of sample EG-PP4. At least 200 particles were analyzed.

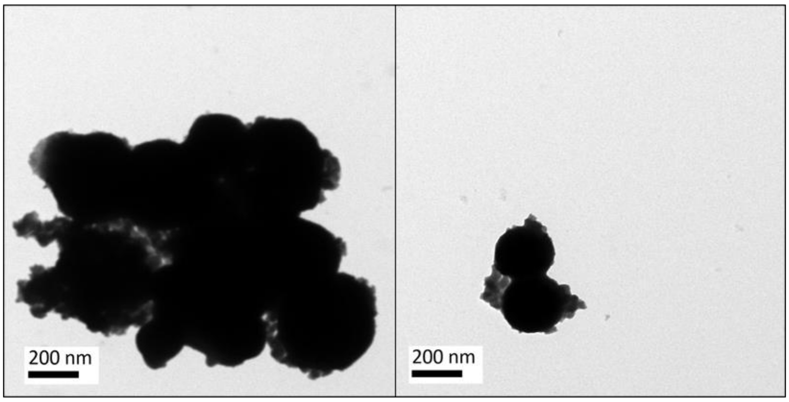

Figure S31. TEM-images of sample EG-MPP4. The quality of the particles was too low to determine the particle sizes.

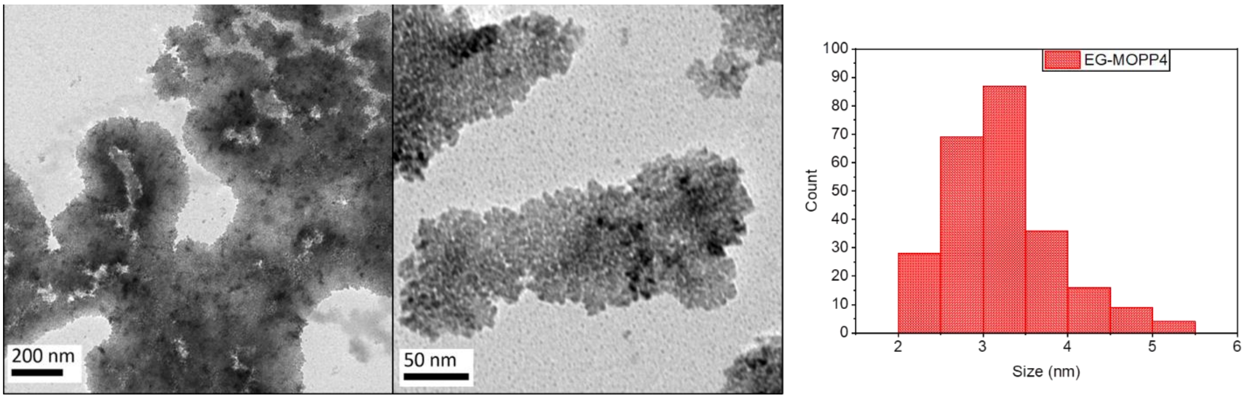

Figure S32. TEM-images and particle-size histogram of sample EG-MOPP4. At least 200 particles were analyzed.

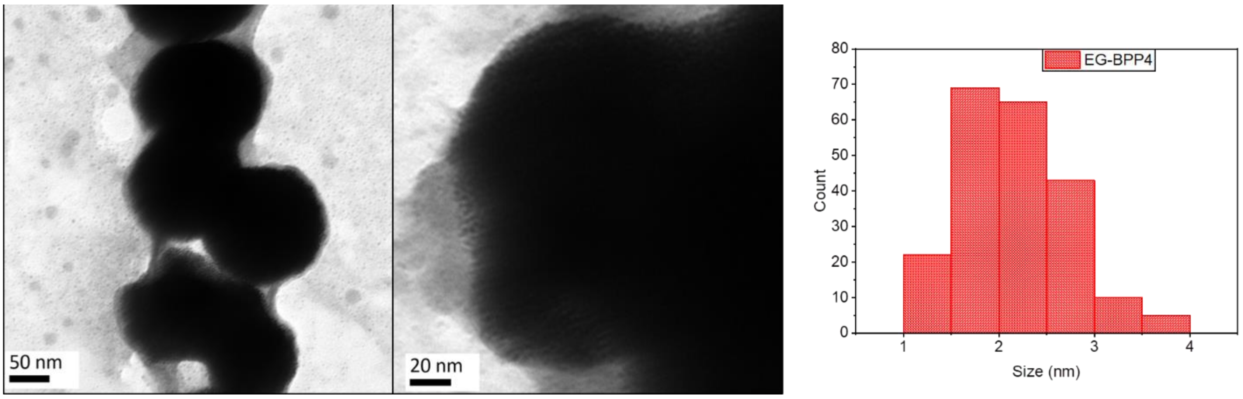

Figure S33. TEM-images and particle-size histogram of sample EG-BPP4. At least 200 particles were analyzed.

S4 Additional electrochemical measurements

Among the (TAAIL)Pt-NP samples, pMP4 shows a low Tafel slope of 24 mV dec^{-1}, despite its high overpotential, and matches Pt/C with 25 mV dec^{-1}. MOPP4 and BPP4 exhibit much higher Tafel slopes of 54 mV dec^{-1} and 81 mV dec^{-1}, respectively.

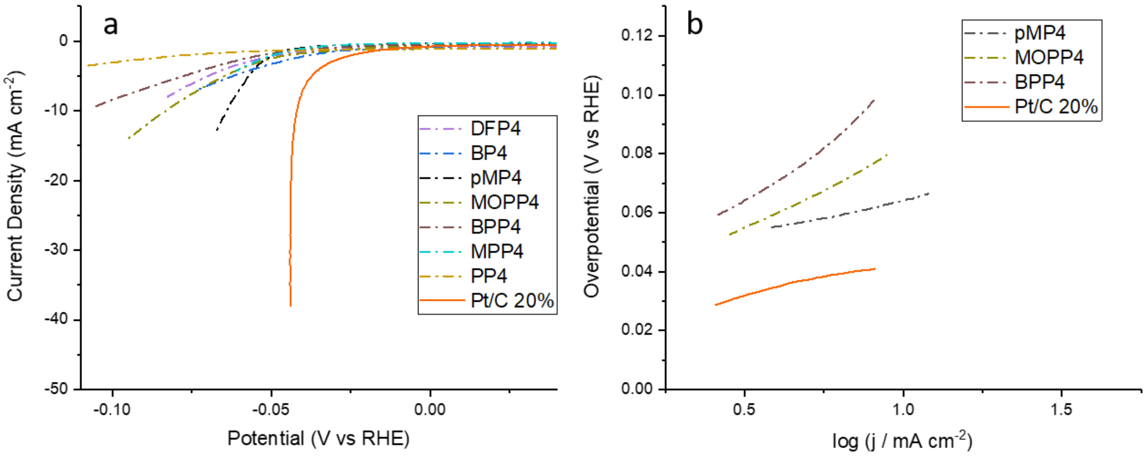

Figure S34. HER polarization curves of (TAAIL)Pt-NPs (**a**) and Tafel plots of samples that reached an overpotential of 10 mA cm^{-2} under the measurement conditions.

S5 Hydrosilylation conversion and product analysis

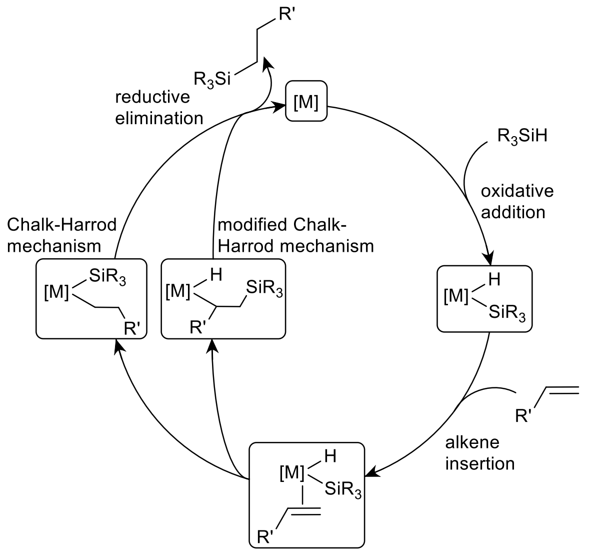

Figure S35. Chalk-Harrod mechanism and a modified Chalk-Harrod mechanism for the transition metal catalysed hydrosilylation of alkenes [3,4]. Reproduced from our previous work [5, Supplementary Materials].

S5.1 Reaction procedure and catalytic activity

Most reactions presented follow the catalysis methods 1 to 3 described in the main text. The reactions serving as references can deviate from these methods as follows:

Method 1: EG/[$Ph_{4\text{-}Br}ImC_4$][NTf_2], EG/[$Ph_{4\text{-}Br}ImPhC_4$][NTf_2] but with no K_2PtCl_6
These reactions have been carried out similar to method 1 without Pt catalyst. 900 mg EG and 100 mg IL and the same amount of educts as mentioned in method 1 have been used for both reactions.

Method 1: ([$Ph_{2\text{-}Me}ImPhC_4$][NTf_2])K_2PtCl_6, ([$Ph_{4\text{-}OMe}ImPhC_4$][NTf_2])K_2PtCl_6, [$Ph_{4\text{-}OMe}ImPhC_4$][NTf_2])$MeCpPtMe_3$ but with no EG
These reactions have been carried out similar to method 1. Instead of EG-IL mixtures, only IL has been used to achieve ~0.2 wt% Pt in IL.

Method 1: K_2PtCl_6 but with no EG and no IL
The reaction has been carried out as one-phase reaction (without EG and IL) neat under the same conditions as mentioned for method 1.

Method 2: MOPP4, BPP4, Pt-NPs but with no EG
These reactions have been carried out as mentioned in method 2 with the respective Pt catalyst.

Method 3: Pt-free, K_2PtCl_6, $MeCpPtMe_3$, Pt-NP but with no EG and no IL
These reactions have been carried out as mentioned in method 3 with the respective Pt catalyst. For the Pt-free sample, the pure educts without any catalyst have been reacted neat.

Table S5. Activity of (IL)Pt-NP samples in the hydrosilylation of phenylacetylene with triethylsilane.

Sample	Molar ratio substrate/Pt [1]	Conversion (%) [2]	Molar ratio distal/proximal from NMR [2,3]	Molar ratio distal/proximal from GC [4]
**Method 1** [5]				
(EG/[$Ph_{4-Me}ImC_4$][NTf_2])K_2PtCl_6	8790	77	3.5	2.9
(EG/[$Ph_{4-OMe}ImC_4$][NTf_2])K_2PtCl_6	8300	77	3.4	3.3
(EG/[$Ph_{4-Br}ImC_4$][NTf_2])K_2PtCl_6	9620	96	3.1	2.4
(EG/[$Ph_{4-Br}ImC_5$][NTf_2])K_2PtCl_6	9620	96	2.5	2.1
(EG/[$Ph_{2,4-F}ImC_4$][NTf_2])K_2PtCl_6	9190	89	3.5	2.9
(EG/[$PhImPhC_4$][NTf_2])K_2PtCl_6	9940	99	3.0	2.3
(EG/[$Ph_{2-Me}ImPhC_4$][NTf_2])K_2PtCl_6	10100	>99	3.1	2.4
(EG/[$Ph_{4-OMe}ImPhC_4$][NTf_2])K_2PtCl_6	8300	98	3.5	2.8
(EG/[$Ph_{4-Br}ImPhC_4$][NTf_2])K_2PtCl_6	8790	>99	2.2	1.8
EG/[$Ph_{4-Br}ImC_4$][NTf_2]	-	0	-	-
EG/[$Ph_{4-Br}ImPhC_4$][NTf_2]	-	0	-	-
([$Ph_{2-Me}ImPhC_4$][NTf_2])K_2PtCl_6	9500	0	-	-
([$Ph_{4-OMe}ImPhC_4$][NTf_2])	9100	0	-	-
([$Ph_{4-OMe}ImPhC_4$][NTf_2])$MeCpPtMe_3$	7900	<5	-	-
(EG)K_2PtCl_6	9660	50	3.6	-
K_2PtCl_6	8820	0	-	-
**Method 2** [6]				
EG-pMP4	1110	5	-	-
EG-MOP4	1090	29	3.2	2.5
EG-BP4	1050	38	3.6	2.8
EG-BP5	1050	8	-	-
EG-DFP4	1070	7	-	-
EG-PP4	980	57	3.1	1.7
EG-MPP4	970	0	-	-
EG-MOPP4	1060	0	-	-
EG-BPP4	980	75	3.4	1.4
Pt-NP [7]	1010	<5	-	-
MOPP4	1070	<5	-	-
BPP4	1130	<5	-	-

**Method 3** [8]				
3a) (EG/TAAIL)Pt-NP				
EG-pMP4	1000	99	2.4	2.1
EG-BP4	880	>99	1.5	1.3
EG-BP5	980	67	2.2	1.9
EG-DFP4	920	57	2.8	2.7
EG-PP4	1040	46	2.8	2.5
EG-MPP4	950	>99	2.0	1.5
EG-MOPP4	930	>99	1.9	2.5
EG-BPP4	990	99	1.8	1.5
Pt-free	-	0	-	-
K_2PtCl_6	1260	98	1.9	1.3
Pt-NP[6]	1320	91	1.9	1.6
3b) (TAAIL)Pt-NP				
pMP4	950	>99	2.2	2.2
MOP4	840	99	1.8	1.1
BP4	1030	82	2.6	2.3
BP5	1140	95	2.2	1.7
DFP4	1030	99	2.2	1.9
PP4	880	99	1.5	1.0
MPP4	990	>99	2.0	1.5
MOPP4	1010	98	1.7	1.5
BPP4	1220	30	3.4	2.8
$MeCpPtMe_3$	7660	>99	2.4	1.7

[1] Molar ratio of the substrate phenylacetylene to Pt content, with a molar ratio of 1.0 of triethylsilane to phenylacetylene for all reactions. Pt-contents of $K_2PtCl_6{\cdot}6H_2O$, $MeCpPtMe_3$ and Pt-NP were calculated in correlation to the molar weight and assuming quantitative conversion to Pt-NPs. [2] The statistical error of the distal/proximal product ratio and substrate conversion determined by signal intensities in ^{1}H NMR is roughly 5%. [3] Determined by ^{1}H-NMR spectra from the product solution. Reference signals for the distal and proximal product were taken from the literature [6]. Due to a high noise/signal ratio, values are only calculated if at least 10% product yield could be achieved. [4] Determined from peak areas. Due to a high noise/signal ratio, values are only calculated if at least 10% product yield could be achieved. [5] Substrate/EG-IL (9/1 EG/IL ratio) two-phase reactions, time 15 min, microwave induced heating at 30 W, temperature 110 °C and catalysts dispersed in EG-IL mixtures. 12.5 mmol phenylacetylene and triethylsilane rand 0.61-0.73 mg catalyst were used. [6] One-phase reactions, time 15 min, microwave induced heating at 200 W, temperature 110 °C and Pt-NPs as catalysts. 4.9 mmol phenylacetylene and triethylsilane and 0.88-1.01 mg catalyst were used.[7] Reference Pt-NPs were obtained in the IL [BMIm][NTf_2] after a method already described by us [7]. [8] One-phase reactions, time 5 min, microwave induced heating at 200 W, temperature 200 °C and Pt-NP as catalysts. 4.9 mmol phenylacetylene and triethylsilane and 0.80-1.17 mg catalyst have been used. As exception, 12.5 mmol phenylacetylene and triethylsilane and 0.52 mg catalyst have been used for $MeCpPtMe_3$.

The catalyst stability for the hydrosilylation reaction was examined by reusing the Pt catalysts of each method additional times. The catalyst recycling and re-use is described in the main text, Section 3.4. Unfortunately, all catalyst recycling experiments resulted in large losses of conversion after three consecutive catalysis runs, reaching only up to 20 % of the initial conversion value and most catalyst samples do not show any conversion at all anymore at the first recycling run.

S5.2 NMR analysis

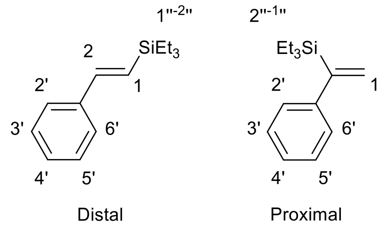

The following NMR values and figures were obtained from the hydrosilylation catalysis with (EG/[Ph$_{2\text{-Me}}$ImPhC$_4$][NTf$_2$])K$_2$PtCl$_6$ method 1 and are shown exemplarily for all experiments. The peaks can be slightly altered for other reactions.

Distal:

^{1}H NMR (300 MHz, CDCl$_3$): δ 7.02 - 7.40 (m, 5H, 2'-6'-H$_5$), 6.82 (d, 1H, ^3J = 19.3 Hz, 2-H), 6.34 (d, 1H, ^3J = 19.3 Hz, 1-H), 0.80 – 0.96 (m, 9H, 3·2''-H$_3$), 0.52 - 0.65 (m, 6H, 3·1''-H$_2$).

^{13}C NMR (75 MHz, CDCl$_3$): δ 145.0, 138.7, 128.6, 128.0, 126.5, 126.0, 7.6, 3.7.

Proximal:

^{1}H NMR (300 MHz, CDCl$_3$): δ 7.02 - 7.40 (m, 5H, 2'-6'-H$_5$), 5.79 (d, 1H, ^3J = 3.1 Hz, 1-H)*, 5.49 (d, 1H, ^3J = 3.1 Hz, 1-H)*, 0.80 – 0.96 (m, 9H, 3·2''-H$_3$), 0.52 - 0.65 (m, 6H, 3·1''-H$_2$); *signal assignment interchangeable [8].

^{13}C NMR (75 MHz, CDCl$_3$): δ 150.6, 145.6, 128.9, 128.2, 126.8, 126.2, 7.4, 3.5.

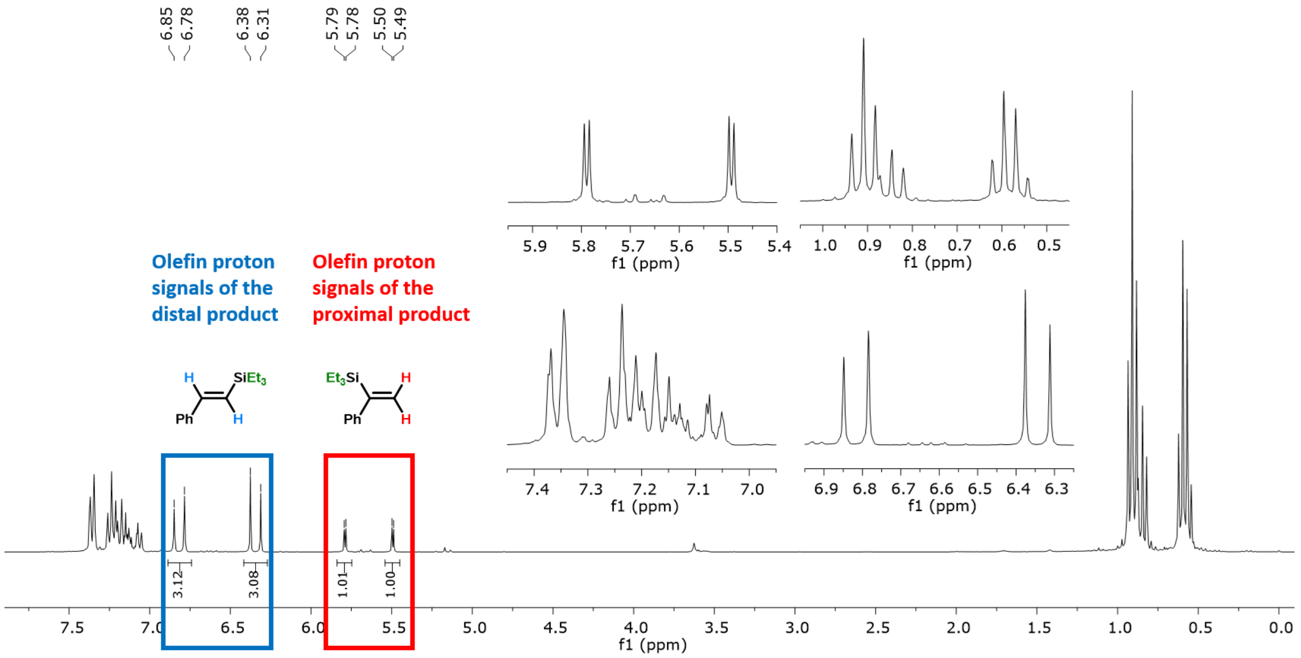

Figure S36. ^{1}H NMR spectrum (300 MHz, CDCl$_3$) with expanded sections of the hydrosilylation product from sample (EG/[Ph$_{2\text{-Me}}$ImPhC$_4$][NTf$_2$])K$_2$PtCl$_6$ with method 1. The conversion was 99% and the molar distal/proximal ratio 3.1.

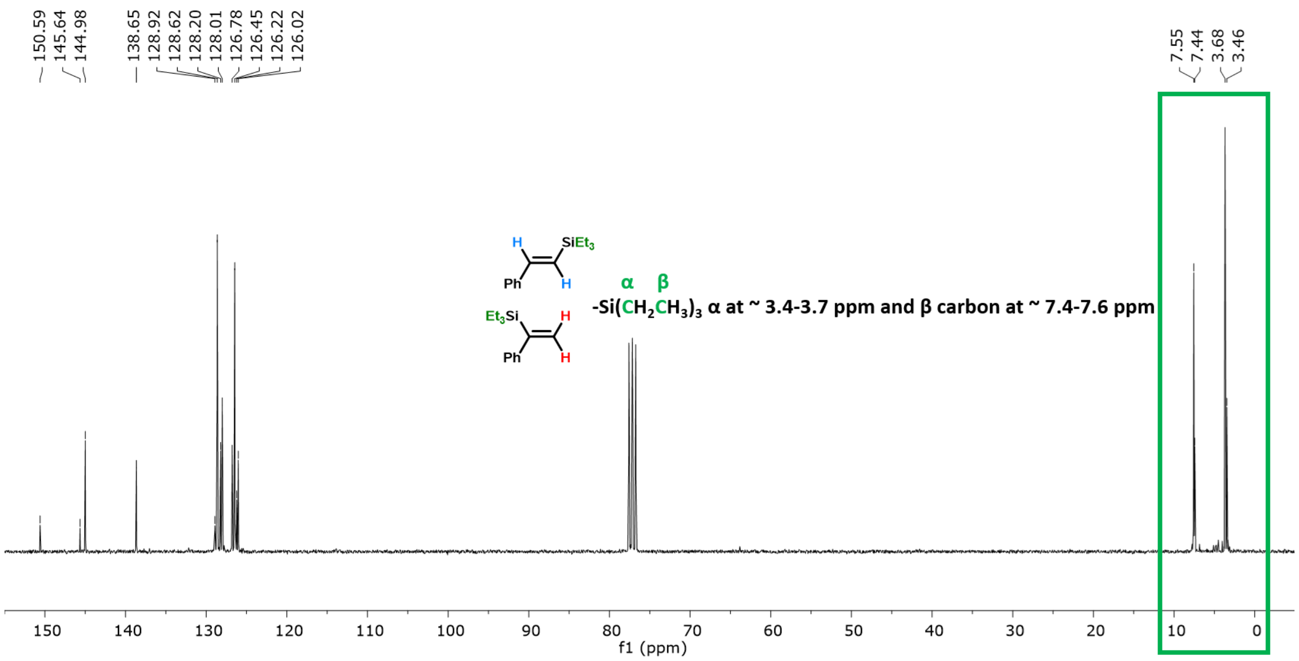

Figure S37. ^{13}C NMR spectrum (75 MHz, $CDCl_3$) of the hydrosilylation product from sample (EG/[$Ph_{2\text{-}Me}$ImPhC_4][NTf_2])K_2PtCl_6 with method 1. Carbon α at ~3.4-3.7 ppm and carbon β at ~7.4-7.6 ppm. The two peaks each for α and β are due to the distal and proximal products.

S5.3 Atomic absorption spectrometry (AAS)

Table S6. Pt atomic absorption spectrometry (AAS) of product solutions after catalysis.

Sample	Pt detected (mg L^{-1}) [1]	Sample	Pt detected (mg L^{-1}) [1]
**Method 1** [2]		**Method 2** [3]	
(EG/[$Ph_{4\text{-}Me}$ImC_4][NTf_2])K_2PtCl_6	20	EG-BP4	28
(EG/[$Ph_{4\text{-}OMe}$ImC_4][NTf_2])K_2PtCl_6	24	EG-BPP4	13
(EG/[$Ph_{4\text{-}Br}$ImC_4][NTf_2])K_2PtCl_6	26	**Method 3** [4]	
(EG/[$Ph_{4\text{-}Br}$ImC_5][NTf_2])K_2PtCl_6	8	*3a) (EG/TAAIL)Pt-NP*	
(EG/[$Ph_{2,4\text{-}F}$ImC_4][NTf_2])K_2PtCl_6	12	EG-BP4	80
(EG/[PhImPhC_4][NTf_2])K_2PtCl_6	5	EG-BPP4	51
(EG/[$Ph_{2\text{-}Me}$ImPhC_4][NTf_2])K_2PtCl_6	13	*3b) (TAAIL)Pt-NP*	
(EG/[$Ph_{4\text{-}OMe}$ImPhC_4][NTf_2])K_2PtCl_6	13	BP4	1.4
(EG/[$Ph_{4\text{-}Br}$ImPhC_4][NTf_2])K_2PtCl_6	2.0	BPP4	1.0

[1] Pt amount detected in 0.2 mL of product solution. The obtained values can deviate within a range of ±10%. [2] Substrate/EG-IL (9/1 EG/IL ratio) two-phase reactions, time 15 min, microwave induced heating at 30 W, temperature 110 °C and catalysts dispersed in EG-IL mixtures. 12.5 mmol phenylacetylene and triethylsilane and 0.61-0.73 mg catalyst were used. [3] One-phase reactions, time 15 min, microwave induced heating at 200 W, temperature 110 °C and Pt-NP as catalysts. 4.9 mmol phenylacetylene and triethylsilane and 0.88-1.01 mg catalyst were used. [4] One-phase reactions, time 5 min, microwave induced heating at 200 W, temperature 200 °C and Pt-NP as catalysts. 4.9 mmol phenylacetylene and triethylsilane and 0.80-1.17 mg catalyst were used.

S5.4 Gas chromatography (GC) analysis

Table S7. Gas chromatography (GC) of product solutions after catalysis. Only samples with at least 10 % product yield are presented.

Sample	Retention time (min)			Sample	Retention time (min)		
	distal product	proximal product	d/p ratio [1]		distal product	proximal product	d/p ratio [1]
**Method 1** [2]				**Method 3** [4]			
(EG/[$Ph_{4\text{-}Me}ImC_4$][NTf_2])-K_2PtCl_6	7.29	6.54	2.9	*3a) (EG/TAAIL)Pt-NPs*			
(EG/[$Ph_{4\text{-}OMe}ImC_4$][NTf_2])-K_2PtCl_6	7.26	6.53	3.3	EG-pMP4	7.27	6.54	2.1
(EG/[$Ph_{4\text{-}Br}ImC_4$][NTf_2])-K_2PtCl_6	7.28	6.54	2.4	EG-BP4	7.28	6.55	1.3
(EG/[$Ph_{4\text{-}Br}ImC_5$][NTf_2])-K_2PtCl_6	7.26	6.54	2.1	EG-BP5	7.26	6.52	1.9
(EG/[$Ph_{2,4\text{-}F}ImC_4$][NTf_2])-K_2PtCl_6	7.25	6.52	2.9	EG-DFP4	7.25	6.52	2.7
(EG/[$PhImPhC_4$][NTf_2])-K_2PtCl_6	7.28	6.54	2.3	EG-PP4	7.26	6.52	2.5
(EG/[$Ph_{2\text{-}Me}ImPhC_4$][NTf_2])-K_2PtCl_6	7.28	6.52	2.4	EG-MPP4	7.27	6.54	1.5
(EG/[$Ph_{4\text{-}OMe}ImPhC_4$][NTf_2])-K_2PtCl_6	7.28	6.54	2.8	EG-MOPP4	7.25	6.52	2.5
(EG/[$Ph_{4\text{-}Br}ImPhC_4$][NTf_2])-K_2PtCl_6	7.28	6.54	1.8	EG-BPP4	7.26	6.54	1.5
**Method 2** [3]				K_2PtCl_6	7.32	6.58	1.3
EG-pMP4	7.24	6.53	3.6	Pt-NP [5]	7.26	6.54	1.6
EG-MOP4	7.28	6.54	2.5	*3b) (TAAIL)Pt-NPs*			
EG-BP4	7.26	6.52	2.8	pMP4	7.28	6.54	2.2
EG-BP5	7.24	6.51	3.4	MOP4	7.27	6.54	1.1
EG-DFP4	7.24	6.51	3.4	BP4	7.25	6.52	2.3
EG-PP4	7.30	6.55	1.7	BP5	7.28	6.54	1.7
EG-MOPP4	7.24	6.51	1.6	DFP4	7.28	6.54	1.9
EG-BPP4	7.31	6.56	1.4	PP4	7.25	6.54	1.0
				MPP4	7.27	6.54	1.5
				MOPP4	7.25	6.54	1.5
				BPP4	7.25	6.53	2.8
				$MeCpPtMe_3$	7.29	6.55	1.7

[1] Determined from peak areas. The statistical error of the distal/proximal product ratio determined by signal intensities in GC is roughly up to 10%. [2] Substrate/EG-IL (9/1 EG/IL ratio) two-phase reactions, time 15 min, microwave induced heating at 30 W, temperature 110 °C and catalysts dispersed in EG-IL mixtures. 12.5 mmol phenylacetylene and triethylsilane and 0.61-0.73 mg catalyst were used. [3] One-phase reactions, time 15 min, microwave induced heating at 200 W, temperature 110 °C and Pt-NP as catalysts. 4.9 mmol phenylacetylene and triethylsilane and 0.88-1.01 mg catalyst were used. [4] One-phase reactions, time 5 min, microwave induced heating at 200 W, temperature 200 °C and Pt-NP as catalysts. 4.9 mmol phenylacetylene and triethylsilane and 0.80-1.17 mg catalyst were used. [5] Reference Pt-NP have been obtained in the IL [BMIm][NTf_2] after a method already described by us [7].

The following figure was obtained from the hydrosilylation catalysis with $(EG/[Ph_{2\text{-}Me}ImPhC_4][NTf_2])K_2PtCl_6$ method 1 and is shown exemplarily for all experiments. The peaks can be slightly altered for other reactions.

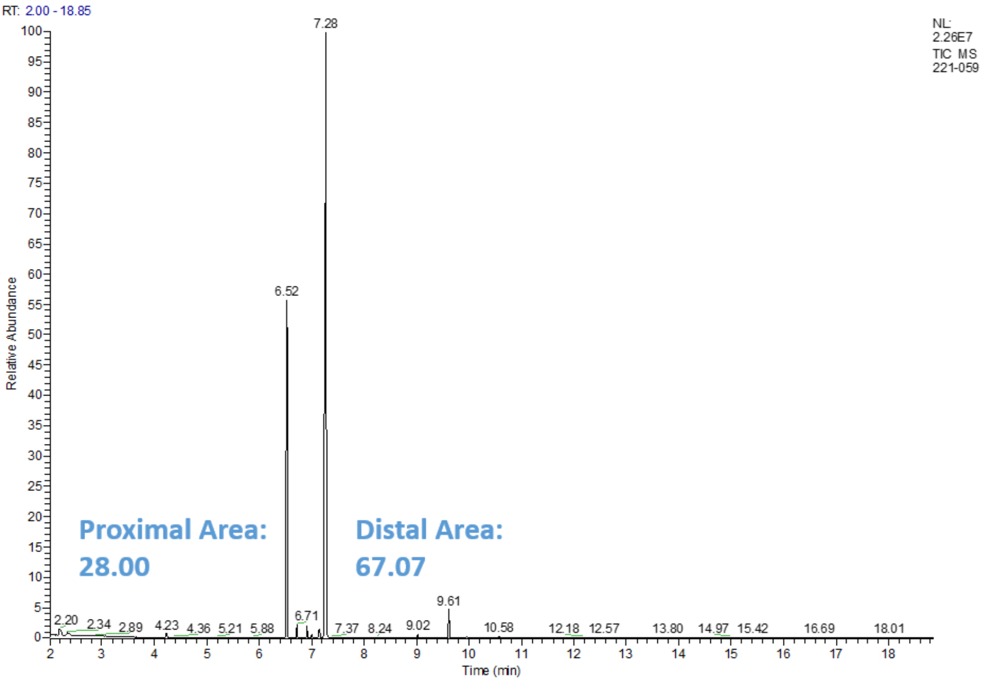

Figure S38. Gas chromatogram of the hydrosilylation product from sample $(EG/[Ph_{2\text{-}Me}ImPhC_4][NTf_2])K_2PtCl_6$ with method 1. The molar distal/proximal ratio was 2.4.

S5.5 TEM-images after catalysis

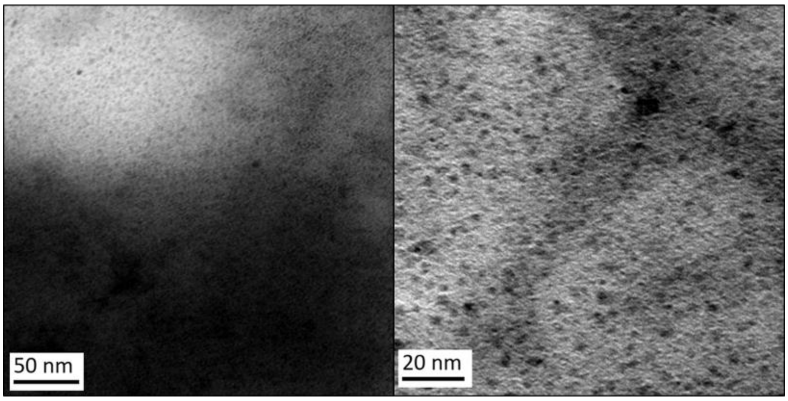

Figure S39. TEM images of sample $(EG/[Ph_{2\text{-}Me}ImPhC_4][NTf_2])$-$K_2PtCl_6$ after the hydrosilylation reaction according to method 1.

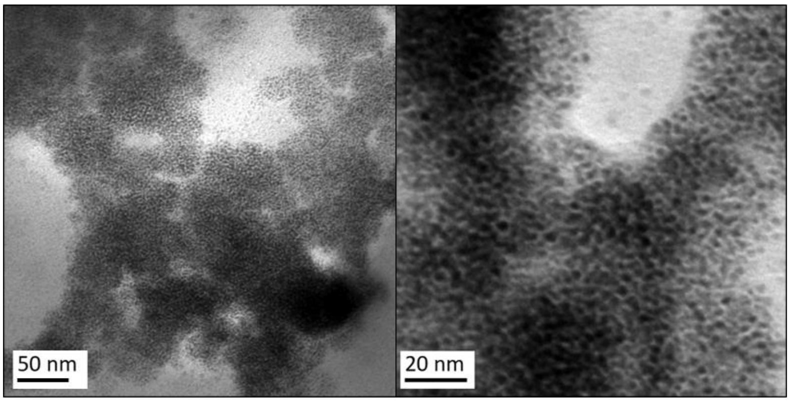

Figure S40. TEM images of sample $(EG/[Ph_{4\text{-}Br}ImPhC_4][NTf_2])$-$K_2PtCl_6$ after the hydrosilylation reaction according to method 1.

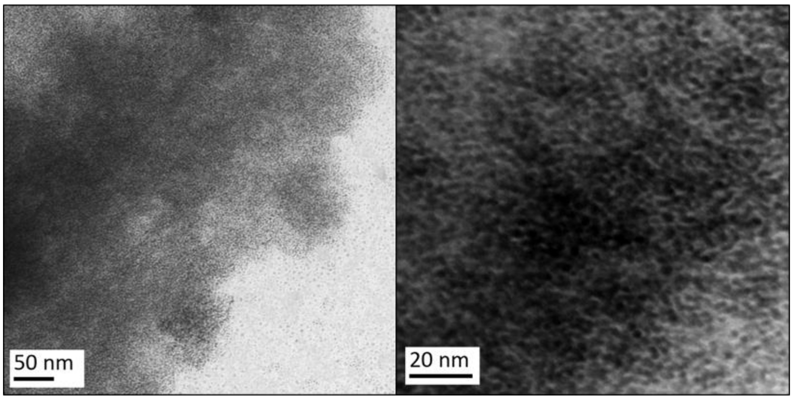

Figure S41. TEM images of sample EG-BPP4 after the hydrosilylation reaction according to method 2.

References

1. Lerch, S.; Strassner, T. Synthesis and Physical Properties of Tunable Aryl Alkyl Ionic Liquids (TAAILs). *Chem. Eur. J.* **2021**, *27*, 15554–15557. https://doi.org/10.1002/chem.202102545.
2. Biller, H.; Strassner, T. Synthesis and Physical Properties of Tunable Aryl Alkyl Ionic Liquids (TAAILs) Comprising Imidazolium Cations Blocked with Methyl-, Propyl- and Phenyl-Groups at the C2 Position. *Chem. Eur. J.* **2022**, e202202795. https://doi.org/10.1002/chem.202202795
3 . Troegel, D.; Stohrer, J. Recent advances and actual challenges in late transition metal catalyzed hydrosilylation of olefins from an industrial point of view. *Coord. Chem. Rev.* **2011**, *255*, 1440–1459. https://doi.org/10.1016/j.ccr.2010.12.025.
4. Chalk, A. J.; Harrod, J. F. Homogeneous Catalysis. II. The Mechanism of the Hydrosilation of Olefins Catalyzed by Group VIII Metal Complexes 1. *J. Am. Chem. Soc.* **1965**, *87*, 16–21. https://doi.org/10.1021/ja01079a004.
5. Woitassek, D.; Moya-Cancino, J. G.; Sun, Y.; Song, Y.; Woschko, D.; Roitsch, S.; Janiak, C. Sweet, Sugar-Coated Hierarchical Platinum Nanostructures for Easy Support, Heterogenization and Separation. *Chemistry* **2022**, *4*, 1147–1160. https://doi.org/10.3390/chemistry4040078.
6. Yong, L.; Kirleis, K.; Butenschön, H. Stereodivergent Formation of Alkenylsilanes:syn oranti Hydrosilylation of Alkynes Catalyzed by a Cyclopentadienylcobalt(I) Chelate Bearing a Pendant Phosphane Tether. *Adv. Synth. Catal.* **2006**, *348*, 833–836. https://doi.org/10.1002/adsc.200606028.
7. Woitassek, D.; Lerch, S.; Jiang, W.; Shviro, M.; Roitsch, S.; Strassner, T.; Janiak, C. The Facile Deposition of Pt Nanoparticles on Reduced Graphite Oxide in Tunable Aryl Alkyl Ionic Liquids for ORR Catalysts. *Molecules* **2022**, *27*, 1018. https://doi.org/10.3390/molecules27031018.
8. Chauhan, M.; Hauck, B. J.; Keller, L. P.; Boudjouk, P. Hydrosilylation of alkynes catalyzed by platinum on carbon. *J. Organomet. Chem.* **2002**, *645*, 1–13. https://doi.org/10.1016/S0022-328X(01)01103-2.
